# Supplementary material for: Tumor suppressor mediated ubiquitylation of hnRNPK is a barrier to oncogenic translation
Source: Nat Commun. 2022 Nov 3;13:6614. doi: 10.1038/s41467-022-34402-6 (PMC9633729; doi:10.1038/s41467-022-34402-6)
Supplement: Supplementary file 1 — Supplementary Information [file 41467_2022_34402_MOESM1_ESM.pdf]

Supplementary Figure 1 hnRNPK is a SCF^{Fbxo4} target.

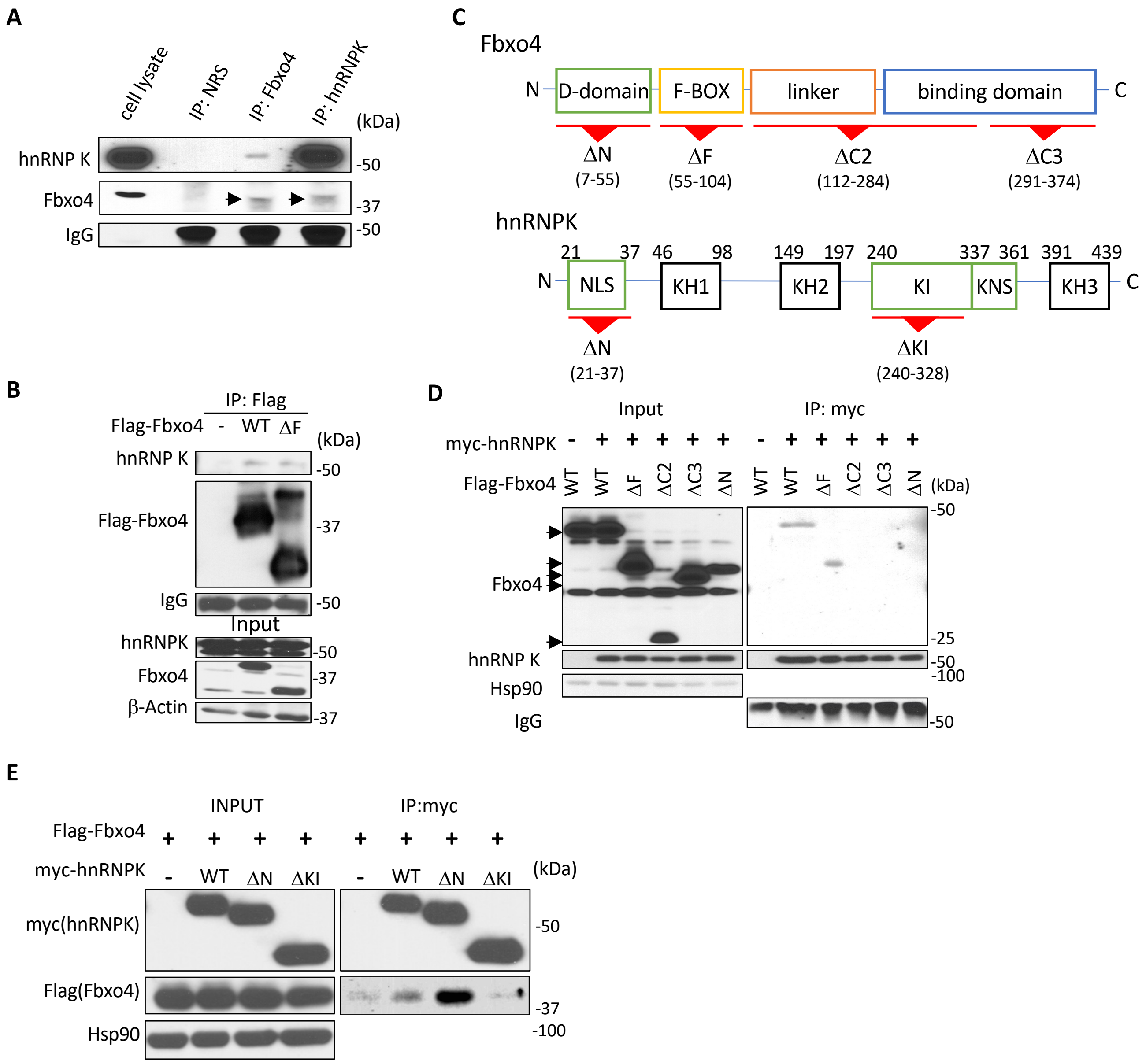

Supplementary Figure 1 hnRNPK is a SCF^{Fbxo4} target.

(**A**) Endogenous hnRNPK and Fbxo4 co-immunoprecipitate in U2OS cells. (**B**) hnRNPK co-immunoprecipitates with ectopic wt Fbxo4 and ΔF Fbxo4- a catalytically deficient mutant unable to recruit E2 ligase but capable of substrate recognition. (**C**) Schematic representation of hnRNPK: K-homology domains 1 – 3; KH1, KH2, KH3), K interacting domain (KI) and K nuclear shuttling (KNC) nuclear localization signal (NLS) and Fbxo4: Fbxo4 dimerization domain (D-domain), F-Box cassette, linker region, substrate binding domain) domain structures with indication of mutants utilized to determined protein recognition regions. (**D, E**) Truncation of c-terminal Fbxo4 or hnRNPK KI domain deletion impairs Fbxo4-hnRNPK binding. N-terminal truncations shows impaired substrate recognition due to lack of D-domain mediated Fbxo4 dimerization required for SCF-Fbxo4 activity.

Supplementary Figure 2 SCF^{Fbxo4} catalyzes hnRNPK ubiquitylation in vivo and in vitro

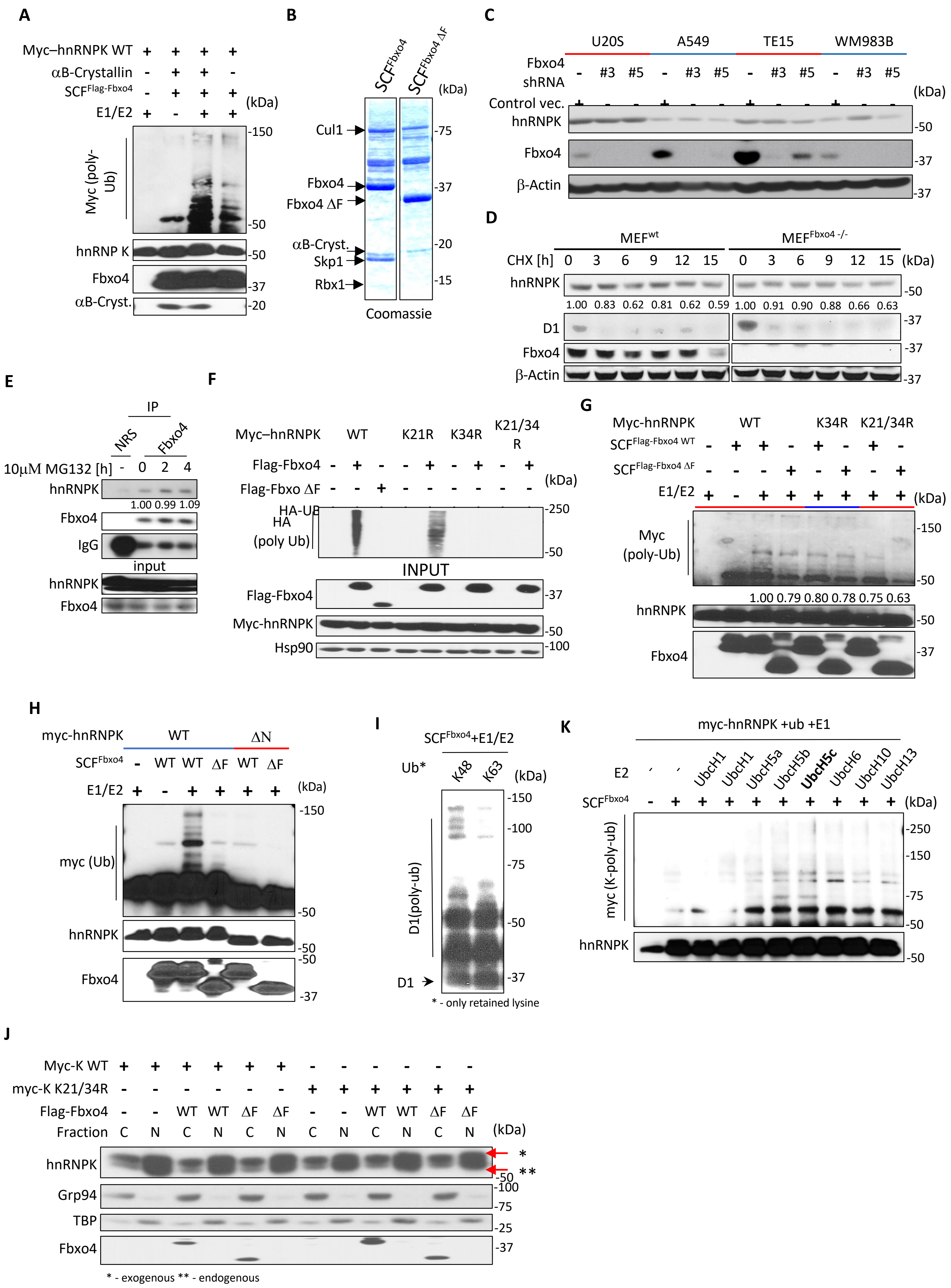

Supplementary Figure 2 SCF^{Fbxo4} catalyzes hnRNPK ubiquitylation *in vivo* and *in vitro*

(**A**) *In vitro* ubiquitylation of hnRNPK by SCF^{Fbxo4} is αB-Crystallin dependent. (**B**) Coomassie blue stain of SCF^{Fbxo4} complex reconstituted and purified from Sf9 insect cells. (**C**)The effect of shRNA-mediated Fbxo4 knock-down in melanoma (WM983B), osteosarcoma (U2OS), lung cancer (A549) and ESCC (TE15) cell lines on hnRNPK protein levels. (**D**) The effect of Fbxo4 deficiency in MEFs on hnRNPK protein levels and $t_{1/2}$ measuerd by CHX chase (cyclin D1- positive control). Band intensity of hnRNKP was corrected to loading control and compared to 0 min time point (**E**) Coimmunoprecipitation of endogenous Fbxo4 and hnRNPK in HEK293T cells with or without treatment with 26S proteasome inhibitor MG132 (10μM); signal intensity of hnRNPK was corrected to Fbxo4 (IP) and compared to 0 min time point (**F, G**) Lys-21 and Lys-34 of hnRNPK are essential for SCF^{Fbxo4} driven hnRNPK polyubiquitylation *in vivo* (F) and *in vitro* (G); ubiquitylation signal in (G) was quantified by average intensity and compared to signal from lane 3. (**H**) *in vitro* ubiquitylation assay with the use of wt or N-terminal truncated hnRNPK. (**I**) SCF^{Fbxo4} elongates polyubiquitin conjugates on cyclin D1 preferentially through K48 in *in vitro* ubiquitylation assay. (**J**) The effect of SCF^{Fbxo4} on hnRNPK cytoplasmic/nuclear distribution was evaluated by fractionation of HEK293T cells; wt of K21/34R myc-hnRNPK mutant was co-overexpressed with either wt or catalytically inactive ΔF Flag-Fbxo4. (**K**) A set of E2 ligases was screen for efficient ubiquitylation of hnRNPK *in vitro*; UbcH5c (bolded) was utilized in all ubiquitylation experiments.

Supplementary Figure 3 SCF^{Fbxo4}–hnRNPK controls c-Myc synthesis and affects the genomic landscape.

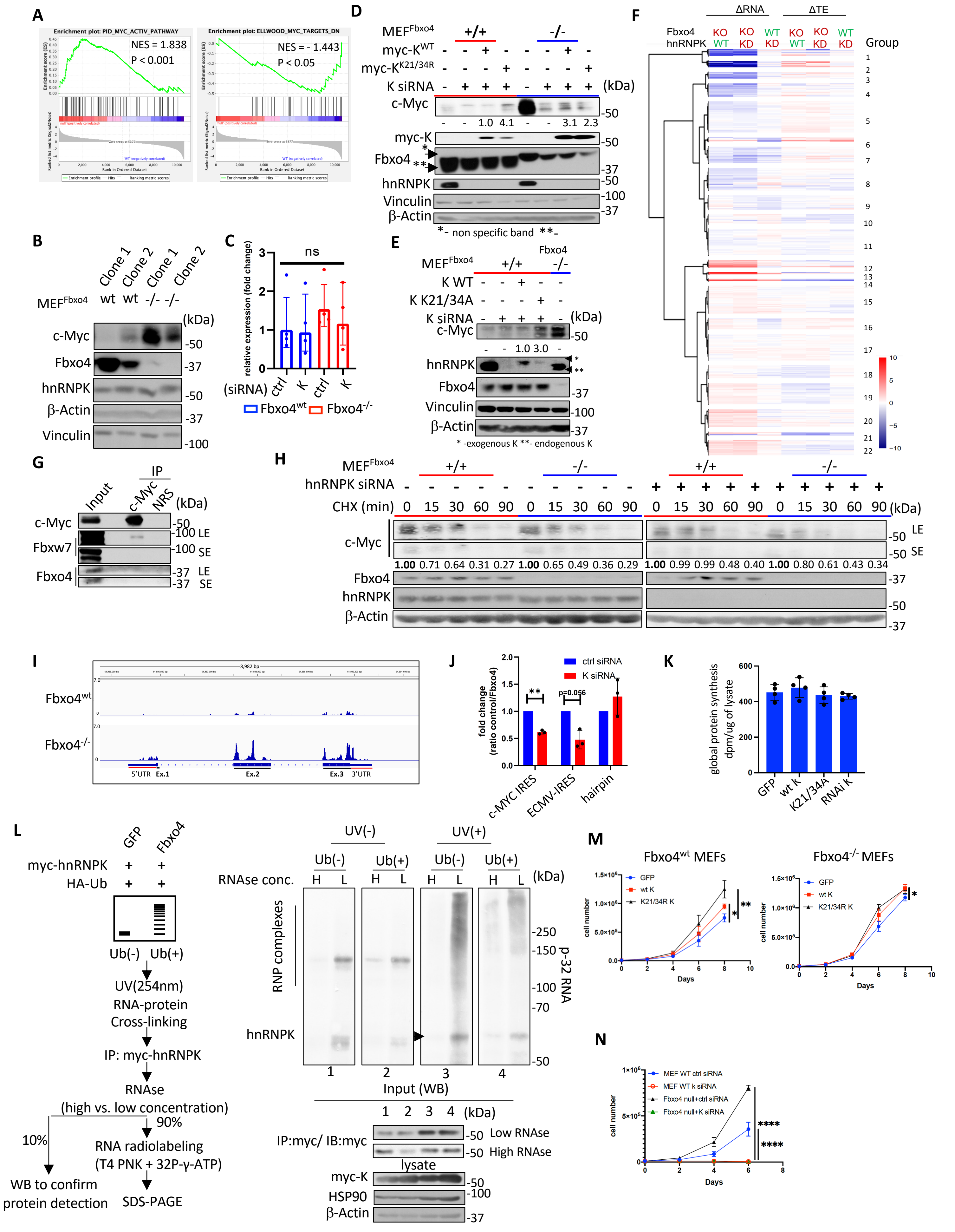

Supplementary Figure 3 SCF^{Fbxo4}–hnRNPK controls c-Myc synthesis and affects the genomic landscape.

(**A**) GSEA analysis of mRNA dysregulated in Fbxo4 null cells reveals c-Myc signature. (**B**) c-Myc expression in different MEF^{Fbxo4}/MEF$^{Fbxo4-/-}$ clones. (**C**) qPCR analysis of c-Myc mRNA in MEF^{Fbxo4}/MEF$^{Fbxo4-/-}$ with or without hnRNPK knock-down. (**D, E**) Depletion of endogenous hnRNPK followed by retroviral transduction of different (K21/34R or K21/34A) ubiquitin mutant hnRNPK results in upregulation of c-Myc. (**F**) RNAseq and RiboSeq performed on the following conditions MEF^{wt}, MEF$^{Fbxo4\ -/-}$, MEF$^{wt\ +\ hnRNPK\ RNAi}$, MEF$^{Fbxo4-/-\ +\ hnRNPK\ RNAi}$ summarized as heatmap. (**G**) Previously established c-Myc recognizing F-box protein Fbxw7 co-purifies with c-Myc but not Fbxo4 in U2OS cells. (**H)** c-Myc decay rate in CHX chase assay upon Fbxo4/hnRNPK modification; c-Myc western blot has been quantified with correction to β-Actin blot (**I**) Ribosome coverage (RPF) of c-Myc transcript in Fbxo4$^{-/-}$ vs Fbxo4^{wt} cells. (**J**) c-MYC IRES activity in dual luciferase, bi-cistronic reporter assay upon hnRNPK knock-down (ECMV IRES- luciferase signal positive control; hairpin - negative control). (**K**) Measured by ^{35}S incorporation assay global translation rate in MEFs (n=4) expressed as dpm (disintegration rate per minute) per µg of protein lysate. (**L**) *In vivo* ubiquitylation of hnRNPK in HEK293T cells followed by UV cross linking and immunoprecipitation was utilized to determine hnRNPK binding affinity to RNA, two concentrations (H- high, L- low) of RNAse were applied to validate presence of RNA. (**M**) Fbxo4$^{-/-}$ cells exhibit higher proliferation rate; ectopic expression of hnRNPK ubiquitin mutant increase MEF doubling time greater than wt; (**N**) hnRNPK depletion inhibits cell proliferation. Data in C, K, M, N represents mean ±SD and was analyzed by multiple t-test (**M** – n=4, **N** – n=3) or one way ANOVA (**C** – n=4). Data presented in J (n=3) shows mean ratio (control/K siRNA sample) and was compared by paired ratio Student's t-test. *p<0.05, **p<0.01, ****p<0.0001. In **(M)** and **(N)** present statistic comparison from the last time point. Exact p-Values from left to right (C) p=0.6654; (J) p=0.0045, p=0.056; (M) p=0.015, p=0.0082, p=0.0358; (N) p=<0.0001

Supplementary Figure 4 SCF^{Fbxo4} – hnRNPK – c-Myc axis regulates cell invasion and motility.

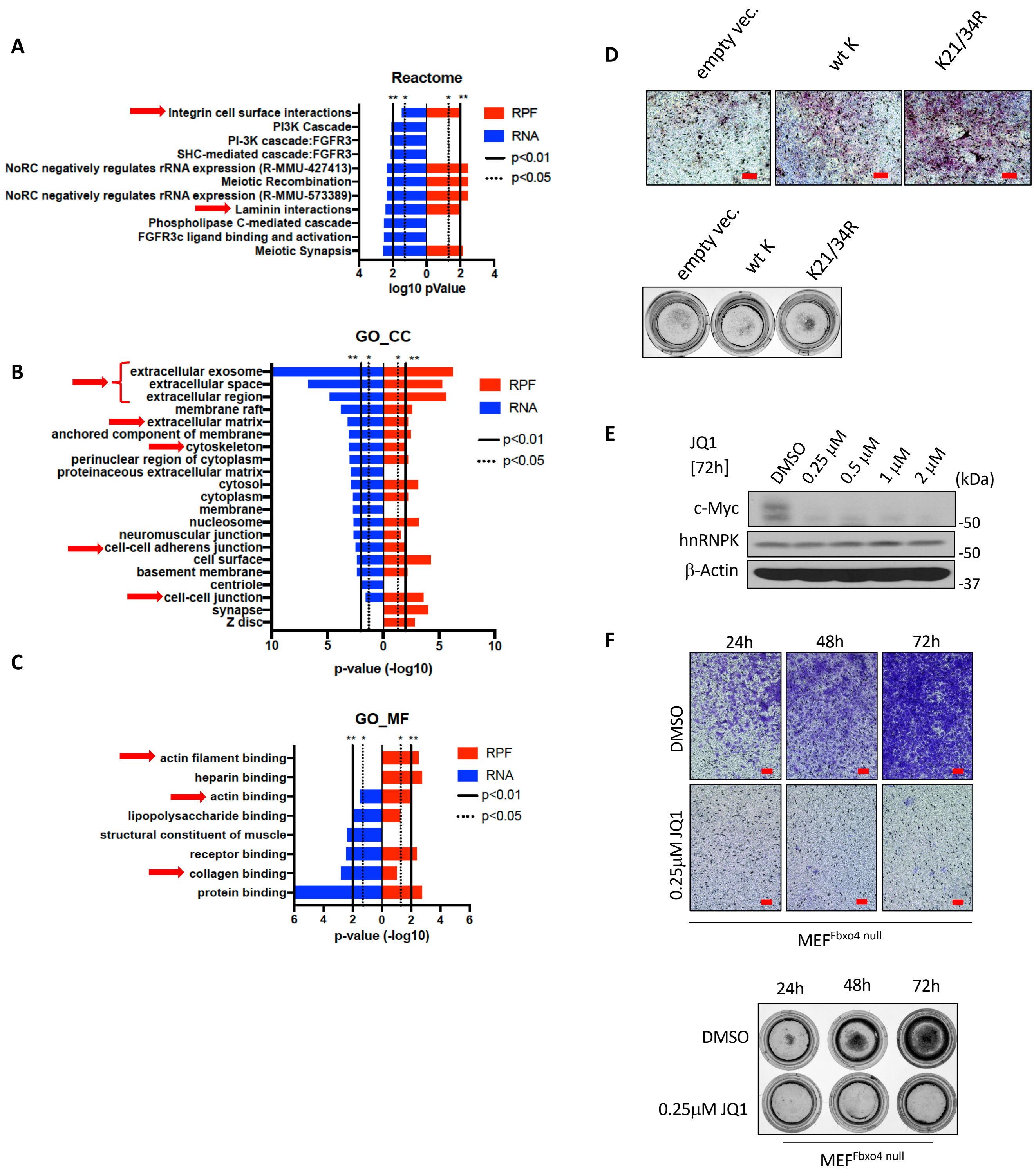

Supplementary Figure 4 SCF^{Fbxo4} – hnRNPK – c-Myc axis regulates cell invasion and motility.

(**A, B, C**) Collection of genes from Reactome, Gene Ontology Cellular Compartment (GO_CC) and Molecular Function (GO_MF) were used to determine the functional meaning of targets identified in Fig 3E, analysis was performed with respect to targets differentially regulated at RNA only, RPF only or both RNA+RPF levels to define potential mechanism. (**D**) Invasion through extracellular matrix of MEF cells transduced with empty vector, wt hnRNPK or ubiquitin mutant. (**E**) NIH3T3 cells were treated with a various concentration of JQ1 (BRD2/4 inhibitor) in order to determine its ability to target c-Myc. (**F**) The effect JQ1 treatment on ability of MEF$^{Fbxo4-/-}$ cells to invade through ECM measured by trans well-assay. Bioinformatic data (**A, B, C**) was evaluated by Fisher's exact test $p < 0.01$ are considered significant. Scale bars - 100μm.

Supplementary Figure 5 SCF^{Fbxo4}-hnRNPK axis contributes to melanoma progression.

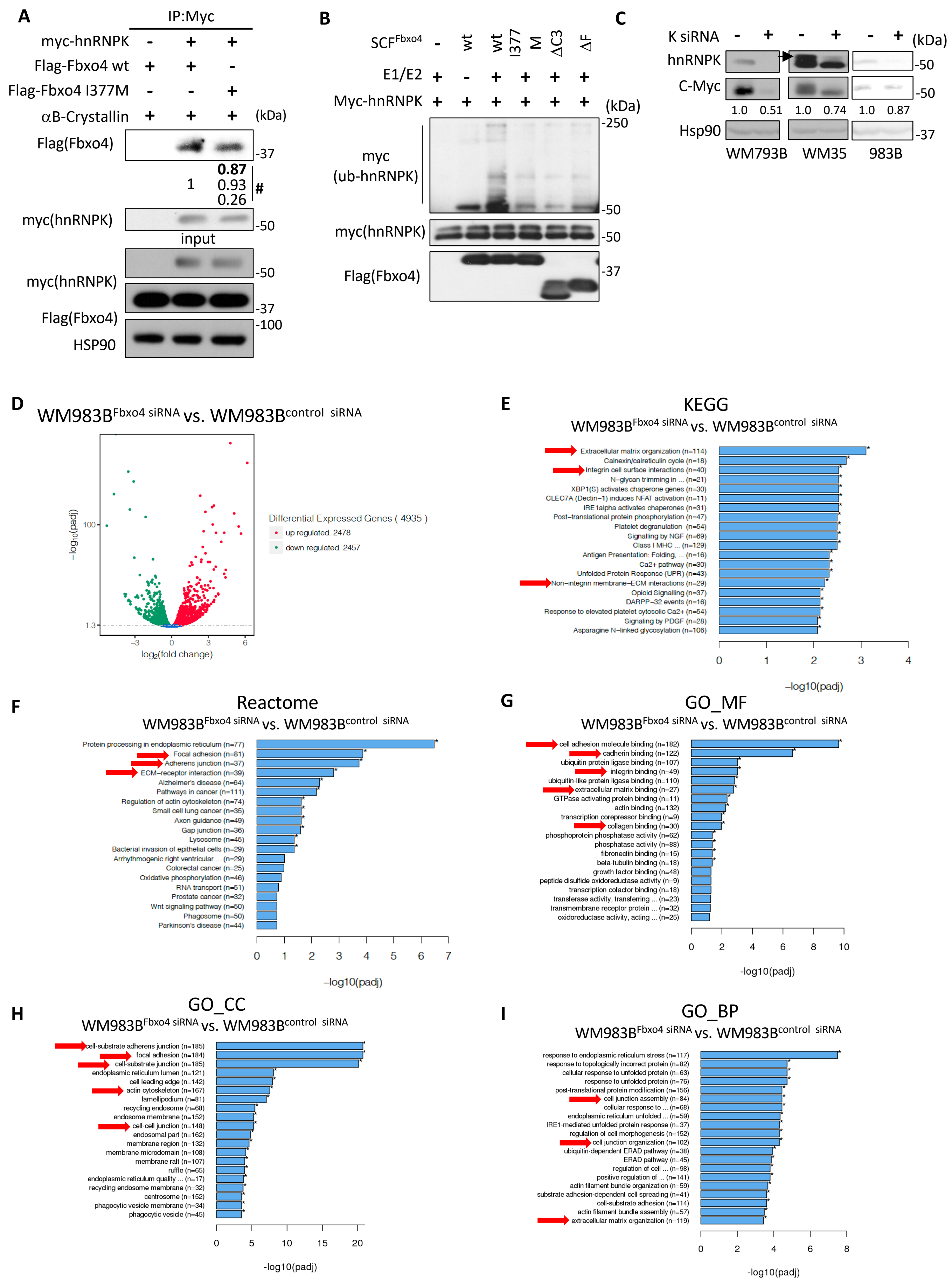

Supplementary Figure 5 SCF^{Fbxo4}-hnRNPK axis contributes to melanoma progression.

(**A**) Fbxo4^{wt} but not Fbxo4^{I377M} co-immunoprecipitates with hnRNPK. (**B**) *In vitro* ubiquitylation assay was used to evaluate the functional consequences of Fbxo4^{I377M} mutation on hnRNPK polyubiquitylation. (**C**) Depletion of hnRNPK in melanoma cell lines downregulates c-Myc. Average intensity of c-Myc western blot has been corrected to Hsp90 (**D**) RNA extracted from WM983B melanoma with Fbxo4 knock-down and control cells were subjected to RNAseq (n=3) which revealed ~5000 differentially regulated genes, differential expression analysis of two conditions/groups was performed using the DESeq2 R package using a model based on the negative binomial distribution, p-Values were adjusted using the Benjamini and Hochberg's approach for controlling the false discovery rate. (**E-I**) Genes identified in (D) were used for functional modeling by Reactome, KEGG, Gene Ontology: Molecular Function (GO_MF), Cellular Compartment (GO_CC), Biological Process (GO_BP) resource, clusterProfiler R package were used to test the statistical enrichment of differential expression genes. *- corrected p-Value<0.05 (padj). # - quantification of three independent experiments expressed as a fold change relative to wt Fbxo4, bolded number refers to data displayed in the figure.

Supplementary Figure 6 part 1 SCF^{Fbxo4}-hnRNPK regulation is impaired in human cancers. (part 1)

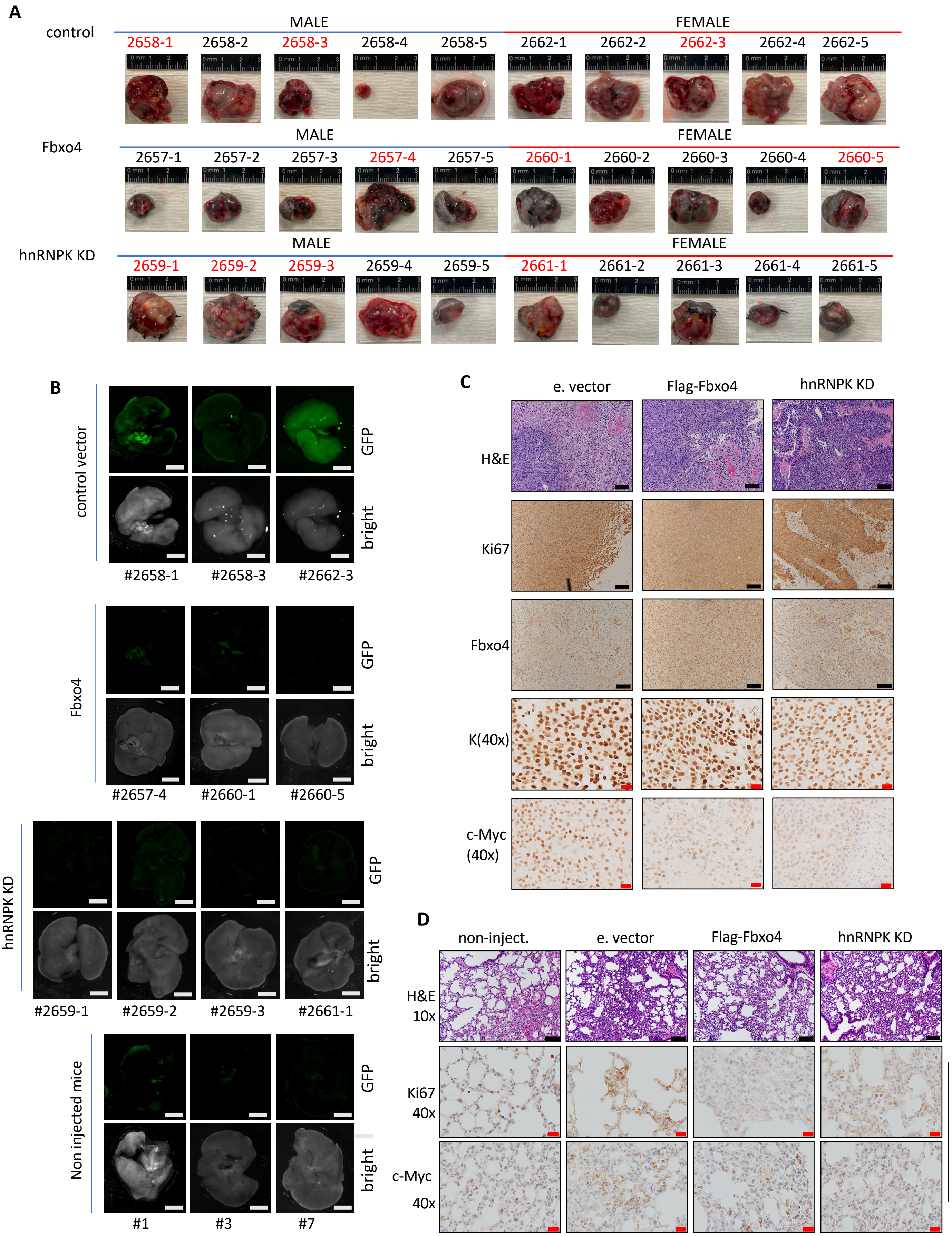

Supplementary Figure 6 part 2 SCF^{Fbxo4}-hnRNPK regulation is impaired in human cancers. (part 2)

E

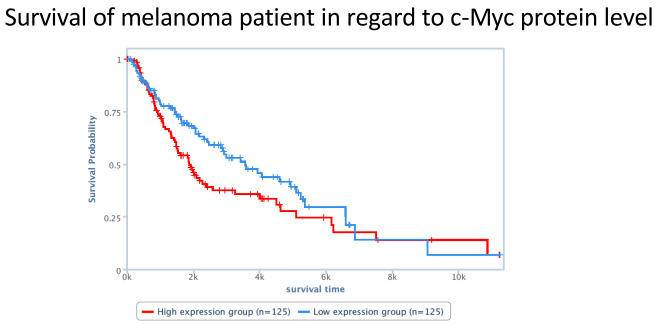

F

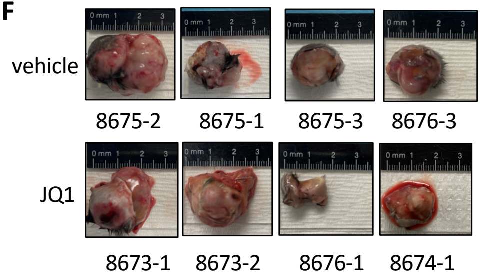

G

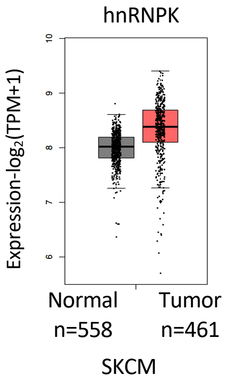

H

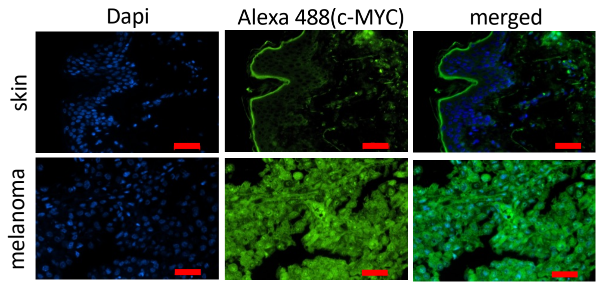

I

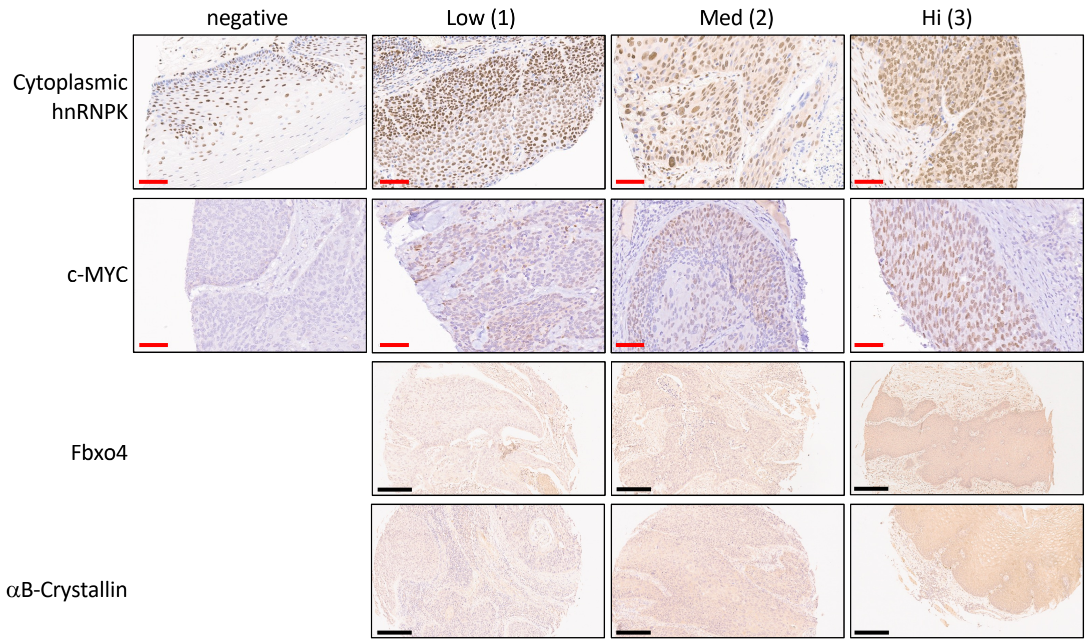

Supplementary Figure 6 SCF^{Fbxo4}-hnRNPK regulation is impaired in human cancers.

(**A**) Images of all tumors harvested post-mortem from B16F10 melanoma allograft model, red labels indicate identification numbers of mice with similar tumor size selected for lung colonization evaluation presented in (**B**) GFP signal from lung were normalized to signal from non-injected mice lungs. (**C**) Immunohistochemical staining of primary tumors to confirm overexpression of Fbxo4, knock-down of hnRNPK and its effect on c-Myc level. (**D**) Example of histological landscape in the lung extracted from B16F10 subcutaneously injected allografts. (**E**) The cancer proteome atlas data (tcpaportal.org: TCGA-SKCM) shows lower survival probability for group of melanoma patients with high c-Myc protein level (n= 125 high expression/125 low expression; Log-Rang $P < 0.05$). (**F**) Images of all tumors extracted in B16F10 melanoma allograft model with JQ1 inhibitor treatment. (**G**). hnRNPK expression in TCGA SKCM (n = 558) compared to the normal (n = 461, TCGA and GTEX) was generated in the GEPIA2 tool. Both groups were compared by one-way ANOVA. Boxplot center represents median, bounds represent 25 and 75%, and whiskers show the minimum or maximum no further than 1.5 times interquartile range from the bound (**H**) Examples of immunofluorescent staining of melanoma TMA; samples were stained with DAPI and c-Myc specific antibody conjugated to Alexa488. (**I**) Representative images for each score in immunohistochemical staining of Fbxo4, αB-Crystallin, cytoplasmic hnRNPK and c-Myc staining. Scale bars: **C**, **D, H** red - 200μm; black - 50μm; **I** red - 60μm, black - 200μm.

Supplementary Figure 7 Graphical summary of working model

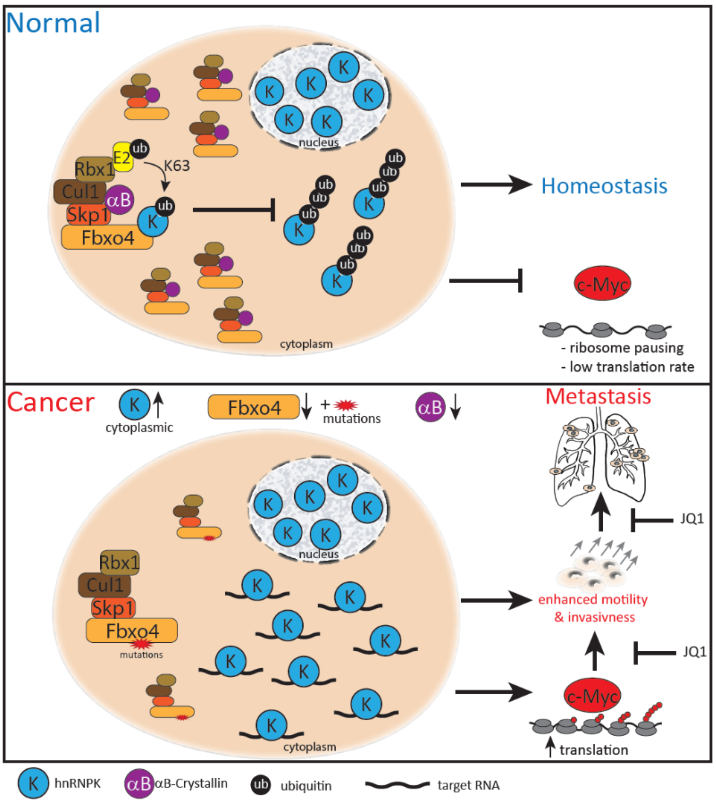

Supplementary Figure 7 Graphical summary of working model

Under normal conditions, cytoplasmic hnRNPK activity is limited by SCF^{Fbxo4} dependent ubiquitylation. In cancer, loss of SCF^{Fbxo4} function in combination with aberrant accumulation of cytosolic hnRNPK leads to a high rate of c-Myc synthesis and dysregulation of pathways involved in cell anchorage, invasion and motility. Loss of Fbxo4 permits hnRNPK to contribute to the metastatic phenotype through enhanced translation of pro-oncogenic factor mRNAs.

Supplementary Figure 8 Replicates correlation of RNA and Ribo-seq

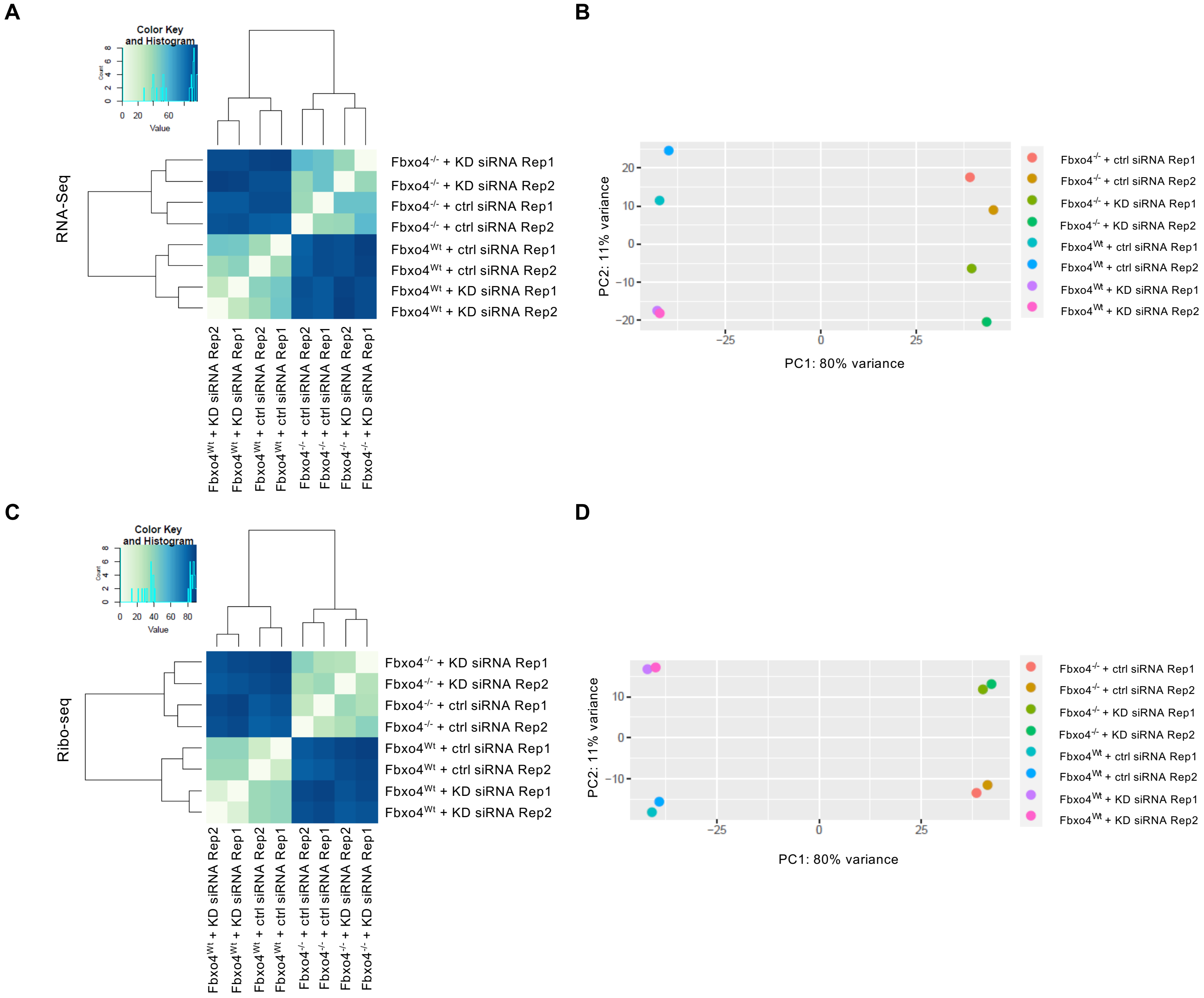

Supplementary Figure 8 Replicates correlation of RNA and Ribo-seq

(**A**) RNA-seq library heatmap of Z-score correlations of each condition with replicates. The scale blue to white indicated increasing similarity, with white being perfect correlation. (**B**) RNA-seq library Principal Component Analysis (PCA) of each condition with replicates. (**C**) Ribo-seq library heatmap of z-score correlations of each condition with replicates. (**D**) Ribo-seq library Principal Component Analysis (PCA) of each condition with replicates.

Supplementary Figure 9 Normalized read counts per mRNA feature

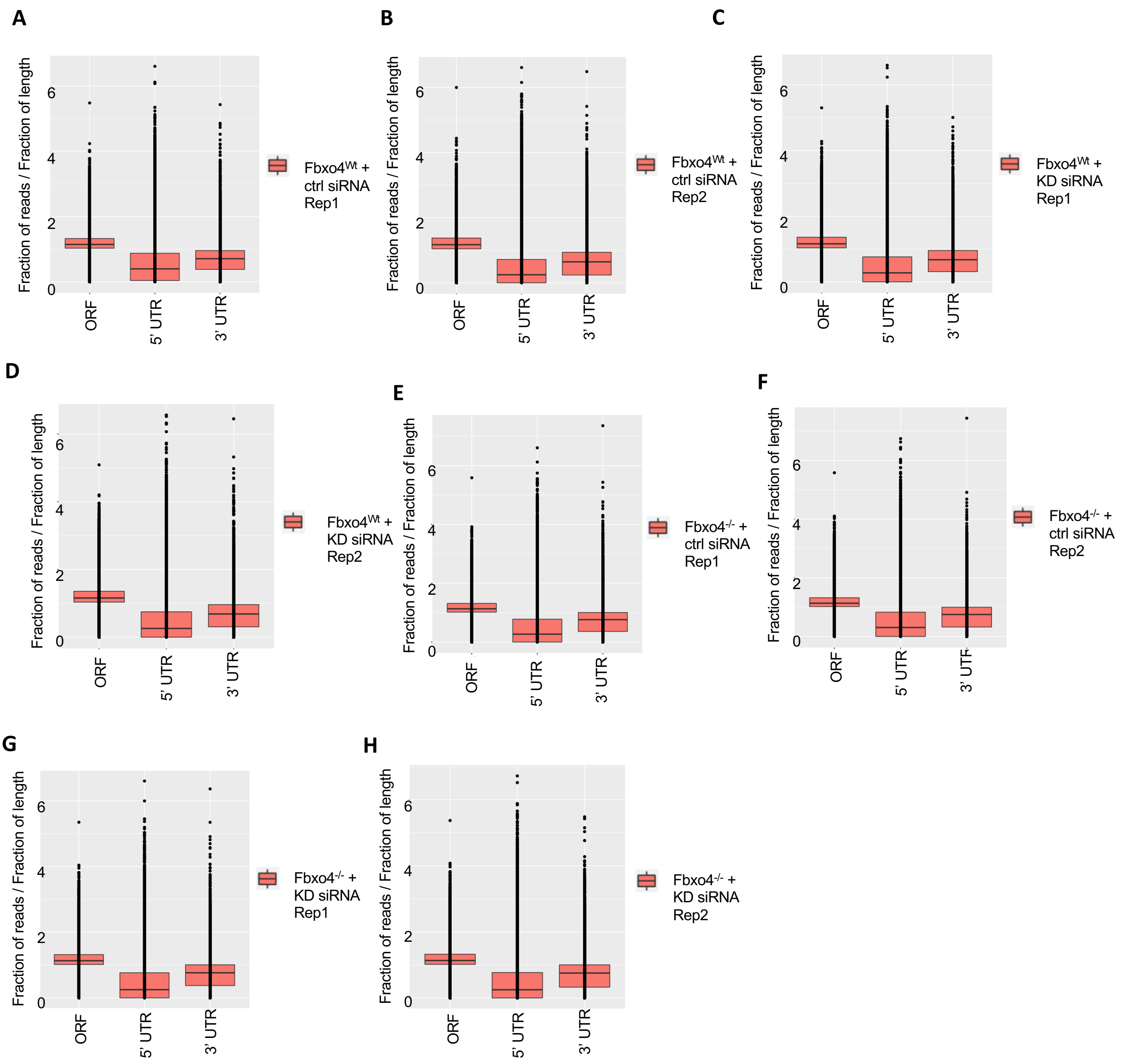

I

Sample	P_VALUE from t-test (two-sided) CDSvs5UTR	CDSvs3UTR	5UTRvs3 UTR
$Fbxo4^{Wt}$ + ctrl siRNA Rep1	<2.2e-16	<2.2e-16	2.693E-10
$Fbxo4^{Wt}$ + ctrl siRNA Rep2	<2.2e-16	<2.2e-16	0.08264
$Fbxo4^{Wt}$ + KD siRNA Rep1	<2.2e-16	<2.2e-16	0.001437
$Fbxo4^{Wt}$ + KD siRNA Rep2	<2.2e-16	<2.2e-16	0.1093
$Fbxo4^{-/-}$ + ctrl siRNA Rep1	<2.2e-16	<2.2e-16	0.7244
$Fbxo4^{-/-}$ + ctrl siRNA Rep2	<2.2e-16	<2.2e-16	9.726E-10
$Fbxo4^{-/-}$ + KD siRNA Rep1	<2.2e-16	<2.2e-16	7.59E-06
$Fbxo4^{-/-}$ + KD siRNA Rep1	<2.2e-16	<2.2e-16	0.979

Supplementary Figure 9 Normalized read counts per mRNA feature. (**A-H**) The fraction of reads per mRNA feature (ORF, 5' UTR, or 3' UTR) normalized to the fraction of the length of that feature compared to the total length (RPF/RNA read density). Box plots shown as the log2. The bottom, middle, and top of the box represents the 25th, 50th (median), and 75th quartiles respectively. Top of the upper whisker represents the maximum value of the data that is within 1.5 times the interquartile range over the 75th percentile. The bottom of the lower whisker represents the minimum value of the data that is within 1.5 times the interquartile range over the 25th percentile. The interquartile range is calculated as the difference between the 75th and 25th percentiles. (**I**) p-Values (t-test) for the pairwise comparison between ORF/UTR RPF/RNA read densities. N values for the A – H respectively: (A): 62921; (B): 58692; (C): 61340; (D): 60607; (E): 61052; (F): 60463; (G): 61267; (H): 59846
