# Supplementary material for: Tumor suppressor mediated ubiquitylation of hnRNPK is a barrier to oncogenic translation
Source: Nat Commun. 2022 Nov 3;13:6614. doi: 10.1038/s41467-022-34402-6 (PMC9633729; doi:10.1038/s41467-022-34402-6)
Supplement: Supplementary file 3 — Description of Additional Supplementary Files [file 41467_2022_34402_MOESM3_ESM.pdf]

Description of Additional Supplementary Files

File Name: Supplementary Data 1
Description: Spreadsheet with the list of genes presented in the Fig 3E

File Name: Supplementary Data 2
Description: output file from amionoacid sequence alignment presented in Fig 2E

File Name: Supplementary Data 3
Description: Input and output of oRNAment analysis presented in Fig 1C

File Name: Supplementary Data 4
Description: Input for GSEA presented in Fig 3A and S3A
