# Supplementary material for: Tumor suppressor mediated ubiquitylation of hnRNPK is a barrier to oncogenic translation
Source: Nat Commun. 2022 Nov 3;13:6614. doi: 10.1038/s41467-022-34402-6 (PMC9633729; doi:10.1038/s41467-022-34402-6)
Supplement: Supplementary file 5 — Supplementary Data 2 [file 41467_2022_34402_MOESM5_ESM.pdf]

LALIGN

Home | Contact
Readseq version 2.1.30 (12-May-2010) Readseq version 2.1.30 (12-May-2010)

lalign output for k vs. d1

[EMBnet-Server] Date: Wed Dec 13 16:55:53 2017

```
# bin/lalign36 -E 10.0 -f -12 -g -2 -s data/blosum62.mat 7448.1.seq 7448.2.seq -J -K 5
LALIGN finds non-overlapping local alignments
 version 36.3.5e Nov, 2012(preload8)
Please cite:
 X. Huang and W. Miller (1991) Adv. Appl. Math. 12:373-381

Parameters not available for: data/blosum62.mat: -12/-2
Query: 7448.1.seq
  1>>>k 464 bp - 464 aa
Library: 7448.2.seq
      295 residues in     1 sequences

Statistics: (shuffled [500]) MLE statistics: Lambda= 0.3254;  K=0.1288
 statistics sampled from 1 (1) to 500 sequences
Threshold: E() < 10 score: 23
Algorithm: Smith-Waterman (SSE2, Michael Farrar 2006) (7.2 Nov 2010)
Parameters: data/blosum62.mat matrix (11:-4), open/ext: -12/-2
 Scan time:  0.010

>>d1 295 bp                                               (295 aa)
 Waterman-Eggert score: 29;  16.6 bits; E(1) <  0.76
26.1% identity (56.5% similar) in 23 aa overlap (210-232:251-273)

     210       220       230
k      LDLISESPIKGRAQPYDPNFYDE
       .. . :: ..   :  ::.  .:
d1     IEALLESSLRQAQQNMDPKAAEE
              260       270

>--
 Waterman-Eggert score: 28;  16.1 bits; E(1) <  0.86
28.9% identity (50.0% similar) in 38 aa overlap (165-202:166-203)

          170       180       190       200
k      AKIKELRENTQTTIKLFQECCPHSTDRVVLIGGKPDRV
       .:. : .:: :   :  :      .  : .:.. :. :
d1     SKMPEAEENKQIIRKHAQTFVALCATDVKFISNPPSMV
         170       180       190       200

>--
 Waterman-Eggert score: 28;  16.1 bits; E(1) <  0.86
27.8% identity (83.3% similar) in 18 aa overlap (35-52:130-147)

           40        50
k      RSRNTDEMVELRILLQSK
```

```
       .: . .:......:: .:
d1     NSIRPEELLQMELLLVNK
     130       140

>--
 Waterman-Eggert score: 27;  15.6 bits; E(1) <  0.93
16.7% identity (59.3% similar) in 54 aa overlap (162-215:94-147)

             170       180       190       200       210
k      VKGAKIKELRENTQTTIKLFQECCPHSTDRVVLIGGKPDRVVECIKIILDLISE
       :: .... :  . . . . ..:  : ..... .   .  :  : ... : :...
d1     VKKSRLQLLGATCMFVASKMKETIPLTAEKLCIYTDNSIRPEELLQMELLLVNK
           100       110       120       130       140

>--
 Waterman-Eggert score: 27;  15.6 bits; E(1) <  0.93
50.0% identity (80.0% similar) in 10 aa overlap (22-31:269-278)

              30
k      RPAEDMEEEQ
       . ::. :::.
d1     KAAEEEEEEE
      270



464 residues in 1 query   sequences
295 residues in 1 library sequences
 Scomplib [36.3.5e Nov, 2012(preload8)]
 start: Wed Dec 13 16:55:53 2017 done: Wed Dec 13 16:55:53 2017
 Total Scan time:  0.010 Total Display time:  0.000

Function used was LALIGN [36.3.5e Nov, 2012(preload8)]
```

SIB Swiss Institute of Bioinformatics | Contact
Back to the Top
