# Supplementary material for: Tumor suppressor mediated ubiquitylation of hnRNPK is a barrier to oncogenic translation
Source: Nat Commun. 2022 Nov 3;13:6614. doi: 10.1038/s41467-022-34402-6 (PMC9633729; doi:10.1038/s41467-022-34402-6)
Supplement: Supplementary file 8 — Reporting Summary [file 41467_2022_34402_MOESM8_ESM.pdf]

nature portfolio

Corresponding author(s): J Alan Diehl

Last updated by author(s): 10 / 17 / 2022

Reporting Summary

Nature Portfolio wishes to improve the reproducibility of the work that we publish. This form provides structure for consistency and transparency in reporting. For further information on Nature Portfolio policies, see our Editorial Policies and the Editorial Policy Checklist.

Statistics

For all statistical analyses, confirm that the following items are present in the figure legend, table legend, main text, or Methods section.

n/a	Confirmed	
☐	☒	The exact sample size (*n*) for each experimental group/condition, given as a discrete number and unit of measurement
☐	☒	A statement on whether measurements were taken from distinct samples or whether the same sample was measured repeatedly
☐	☒	The statistical test(s) used AND whether they are one- or two-sided<br>*Only common tests should be described solely by name; describe more complex techniques in the Methods section.*
☐	☒	A description of all covariates tested
☐	☒	A description of any assumptions or corrections, such as tests of normality and adjustment for multiple comparisons
☐	☒	A full description of the statistical parameters including central tendency (e.g. means) or other basic estimates (e.g. regression coefficient) AND variation (e.g. standard deviation) or associated estimates of uncertainty (e.g. confidence intervals)
☐	☒	For null hypothesis testing, the test statistic (e.g. *F*, *t*, *r*) with confidence intervals, effect sizes, degrees of freedom and *P* value noted<br>*Give P values as exact values whenever suitable.*
☒	☐	For Bayesian analysis, information on the choice of priors and Markov chain Monte Carlo settings
☒	☐	For hierarchical and complex designs, identification of the appropriate level for tests and full reporting of outcomes
☒	☐	Estimates of effect sizes (e.g. Cohen's *d*, Pearson's *r*), indicating how they were calculated

Our web collection on statistics for biologists contains articles on many of the points above.

Software and code

Policy information about availability of computer code

Data collection

No software were used for data collection in this manuscript.

Data analysis

The quantification of tissue microarray immunofluorescence experiment were performed with use of ImageJ Fiji v.1.53c. and Phyton 3.10 applying the following code:
STEP1 (Image J): identification of nuclei and intensity measurment

```
from ij import IJ
from ij.plugin.frame import RoiManager
from ij.io import FileSaver
from ij import Prefs
import os

#Provide source path
source_path = r'
dest_path = r'
analyze_list = []

#find channel (GFP and Dapi) pairs
for f in os.listdir(source_path):
	try:
		name, ext = f.split('.')
		name, channel = name.split()
```

```
        except ValueError:
            continue
        if ext == 'jpg' and channel == 'M' and os.path.exists(os.path.join(source_path, name+' D.jpg')):
            analyze_list.append((name, os.path.join(source_path, name+' D.jpg'), os.path.join(source_path, f)))

#Create an output folder
if not os.path.exists(dest_path):
        os.mkdir(dest_path)

#Quantify and save the results as jpg and csv
for name, f1, f2 in analyze_list:
        img1 = IJ.openImage(f1)
        img1.show()
        Prefs.blackBackground = True
        IJ.run(img1, "Make Binary", "");
        IJ.run(img1, "Watershed", "")
        IJ.run(img1, "Fill Holes", "");
        IJ.run(img1, "Analyze Particles...", "  show=Outlines clear add in_situ")

        img2 = IJ.openImage(f2)
        img2.show()
        rm = RoiManager.getInstance()
        IJ.run("From ROI Manager", "");
        rm.runCommand(img2,"Measure");

        fs = FileSaver(img2)
        fs.saveAsJpeg(os.path.join(dest_path, name+'_result.jpg'))
        IJ.saveAs("Results", os.path.join(dest_path, name+'_result.csv'))
        img1.changes = False
        img1.close()
        img2.close()
```

STEP 2 (Phyton)- Random pick of 100 nuclei

```
import os
import csv
import random

MIN_AREA = 1500
source_path = r'

for f in os.listdir(source_path):
    if f.endswith('.csv'):
        output = []
        with open(os.path.join(source_path, f), 'r') as file:
            temp = []
            reader = csv.reader(file)
            output.append(next(reader))
            for row in reader:
                if int(row[1]) >= MIN_AREA:
                    temp.append(row)
            output.extend(random.choices(temp, k=100))
            print(output)

        with open(os.path.join(source_path, f), 'w', newline='') as file:
            writer = csv.writer(file)
            writer.writerows(output)
```

The ribosome profiling libraries underwent single-end sequencing. The resulting Fastq files were processed using FastQC (v0.11.8; RRID:SCR_014583) for QC analysis. The preprocessing was performed based on the QC report using FASTQ Quality Filter module in the FASTX-Toolkit (RRID:SCR_019035) which was used to extract the bases with 99% accuracy based on Q Score. Reads where less than 70% of bases had an accuracy of at least 99%, were removed. The sequences were then collapsed by UMI and Cutadapt (RRID:SCR_011841) was performed to trim 21-nt adaptor before the first and the last 4-nt were trimmed from the reads to remove the UMI. The reads were aligned to mm10 (Genome Reference Consortium Mouse Build 38 (GCA_000001635.2)) using STAR v2.5.3a (RRID:SCR_004463). The generated bam files were processed with RiboProfiling package v1.2.2 (52) and the coverage counts on the coding regions (CDS) were obtained for each sample based on RiboProfiling function modules, TxDb.Mmusculus.UCSC.mm10.knownGene v3.10.0 (Annotation package for TxDb object(s)) and GenomicFeatures package (v1.46.1; (53)) DESeq2 pipeline (version 1.26.0) was performed based on the expressed raw reads for the differential express gene analysis.

Software used for RNAseq of WM983B cells:
Analysis//Software//Version//Parameter//Remarks
Mapping//STAR//v2.6.1//mismatch = 2//Mapping to a reference
Quantification//HTSeq//v0.6.1 -m union// -
Differential Analysis//DESeq2//v2_1.6.3//padj<0.05//For sample with bio-replicate
Differential Analysis//EdgeR//v3.16.5//padj<0.005 and |log2(FoldChange)| > 1//For sample without bio-replicate
Enrichment Analysis//ClusterProfiler//v2.4.3 padj < 0.05//For GO, KEGG, Reactome enrichment analysis.

For manuscripts utilizing custom algorithms or software that are central to the research but not yet described in published literature, software must be made available to editors and reviewers. We strongly encourage code deposition in a community repository (e.g. GitHub). See the Nature Portfolio guidelines for submitting code & software for further information.

Data

Policy information about availability of data

All manuscripts must include a data availability statement. This statement should provide the following information, where applicable:

- Accession codes, unique identifiers, or web links for publicly available datasets
- A description of any restrictions on data availability
- For clinical datasets or third party data, please ensure that the statement adheres to our policy

Total RNA sequencing data of melanoma cell line (WM983B) generated during this study is available at: NCBI Gene Expression Repository: GSE196396. Ribosome profiling data of MEFs generated through the study is available at NCBI Gene Expression Repository: GSE196483

The following cancer databases has been used to collect the data with the use of code implemented in their online tools:
- Gepia2 tool (http://gepia2.cancer-pku.cn)
- NIH Genomic Data Commons Portal v1.28.0 (https://portal.gdc.cancer.gov/)
- Oncomine database (https://www.oncomine.org)
- The Cancer Proteome Atlas (https://tcpaportal.org)
-- The Encyclopedia of RNA Interactomes (https://starbase.sysu.edu.cn/)

All uncropped scans of blots are available with this article.

The Authors declare that all the data supporting the findings of this study are available withing the article, its supplementary information files, source data file and from the corresponding author on reasonable request.

Field-specific reporting

Please select the one below that is the best fit for your research. If you are not sure, read the appropriate sections before making your selection.

☒ Life sciences ☐ Behavioural & social sciences ☐ Ecological, evolutionary & environmental sciences

For a reference copy of the document with all sections, see nature.com/documents/nr-reporting-summary-flat.pdf

Life sciences study design

All studies must disclose on these points even when the disclosure is negative.

Sample size	For all cell-based work sample size was not predetermined, at least three biological replicates were used to provide reproducibility and sufficient biostatistic significance. For mice-based studies, 8 or 30 tumor/lung samples were collected for final analysis; scientifically, this number can produce high confident level statistical results and was determined based on the previous co-author's experience. TMA analysis was performed on commercially purchased material: melanoma (Biomax: ME2081 16 control/88 melanoma T386a 2 control/4 melanoma ESCC (Biomax: ES1505: 50 control/50 ESCC) or de-identified material collected during previous studies (Natsuizaka, Mitsuteru, et al. Nature communications 8.1 (2017): 1-16.) 49 controls/101 ESCC. No statistical method was used to predetermine sample size in human sample TMA studies.
Data exclusions	No data was excluded from the final analysis.
Replication	All attempts at replication were successful. Western blot experiments presented in Fig 2H, D; S3G, H, L; S4E; S5B were performed in two biologically independent replicates. Western blot experiment presented in Fig 1H; 2A-C, F, G; 3B, C, K, L; 4E; 5A; S1A, B, D, E; S2A, C, E, F, G, H, I, J; S3D, B, E; S5A, C were performed in at least three biologically independent repeats. Ribo-seq experiment was performed in biological duplicates. Remaining experiments were run in at least three biologically independent replicates.
Randomization	All the cells and mice were randomly allocated into different groups.
Blinding	The investigators were blinded to group allocation during data collection and/or analysis.

Reporting for specific materials, systems and methods

We require information from authors about some types of materials, experimental systems and methods used in many studies. Here, indicate whether each material, system or method listed is relevant to your study. If you are not sure if a list item applies to your research, read the appropriate section before selecting a response.

Materials & experimental systems

n/a	Involved in the study
☐	☒ Antibodies
☐	☒ Eukaryotic cell lines
☒	☐ Palaeontology and archaeology
☐	☒ Animals and other organisms
☒	☐ Human research participants
☒	☐ Clinical data
☒	☐ Dual use research of concern

Methods

n/a	Involved in the study
☒	☐ ChIP-seq
☒	☐ Flow cytometry
☒	☐ MRI-based neuroimaging

Antibodies

Antibodies used

Antibody//Manufacturer//Catalog#//Method//Dilution
1)c-Myc//Abcam//ab32072//IHC//1:250
2)c-Myc (alexa Fluor488)//Santa Cruz//sc-40 AF488//IF//1:100
3)c-Myc//Cell Signaling//9402S//WB/IP//1:1000 / 2 µg
4)Fbxo4//YenZym//#YZ1779//IB/IP/IHC//1:1000 / 2-4 µg / 1:300
5)Flag//Sigma-Aldrich//F7425-.2MG//WB/IP//1:1000 / 1-4 µg
6)HA(tag)//Sigma-Aldrich//H6908//WB//1:1000
7)His//Abcam//ab137839//IB//1:1000
8)hnRNPK//Abcam//ab52600//IP/WB/IHC//1 µg / 1:20000 / 1:2000
9)Hsp90//Cell Signaling//4874S//IB//1:10 000
10)αB-Crystallin//Enzo LifeSciences//ADI-SPA-222-F//IHC//1:200
11)αB-Crystallin//Enzo LifeSciences//ADI-SPA-223-F//IHC/WB//1:200 / 1:1000
12)myc (9B11 - tag)//Cell Signaling//2276S//IP/WB//1 µg / 1:1000
13)TBP//Cell Signaling//12578S//IB//1:1000
14)Grp94//Cell Signaling//2104S//IB//1:1000
15)β-Actin//Sigma-Aldrich//A5316//IB//1:100 000
16)Normal Rabbit IgG//Cell Signaling//2729S//IP//1-4 µg
17)Ki-67//Abcam//ab15580//IHC//1:5000
18)Vinculin//Cell Signaling//4650S//WB//1:1000
19)Fbxw7//Bethyl Laboratories//A301-720A//WB//1:1000
20)Fbxl8//ThermoScientific//PA5-31380//WB//1:1000
21)TAMRA//Thermo Scientific//MA1-041//WB//1:1000

Validation

The commercial antibodies have relative validation statements indicating they can be used for these species on the manufacturer's website. c-Myc (Fig 4E), hnRNPK (Fig 3B- mouse, S5C- human) antibodies were validated by RNAi dependent depletion encoding mRNA. Flag, Myc (tag) and αB-Crystallin antibodies were validated by over-expression of Flag (i.e. Fig 2D,F, 3C,L, S1D,E), Myc-tagged (Fig 2A,D,F) proteins or αB-Crystallin (Fig 2A, 3C). Fbxl8, Fbxw7, Vinculin we validated in the previous work (Cancer biology & therapy 23.1 (2022): 348-357.). HA antibody was tested by over-expression of HA-fusion protein by manufacturer and its considered as well established product used in over 900 publications (https://www.sigmaaldrich.com). His antibody was validated by us previously (Nat Commun. 2017 Nov 16;8(1):1534.). Grp94 and TBP antibodies detect compartment specific proteins and were validated in the manuscript by cytoplasmic/nuclear fractionation (Fig 2H, S2J). All home-made antibodies were validated in our previous publications: Fbxo4 antibodies (Nat Commun. 2017 Nov 16;8(1):1534.); mouse anti-cyclin D1 antibody (Cancer Cell. 2008 Jul 8;14(1):68-78.). Anti-Tamra antibody was validated by the manufacturer with the use of WB and IP of Tamra-BSA fusion protein, in addition TAMRA validation was published previously (Nature communications 7.1 (2016): 1-16).

Eukaryotic cell lines

Policy information about cell lines

Cell line source(s)

B16F10 melanoma cell lines were purchased from ATCC. HEK293T, U2OS were purchased in 2014 from ATCC. SF9 cells were purchased in 2018 from Gibco. NIH3T3 and A549 cells were purchased from ATCC in 2019 from ATCC. Primary human melanocytes derived from two independent individuals were purchased from ATCC in 2021. TE15 ESCC cells were kindly provided by Dr. Tetsuro Nishihara who established this cell line. Human melanoma cell lines (1205Lu, WM983B, 451Lu, WM3918, and WM35) were kind gift from Dr. Herlyn's lab from the Wistar Institute collection (Philadelphia, PA). MEF cells were derived from Fbxo4+/+ or Fbxo4-/- transgenic mice embryo (B57BL/6 background developed by Vega Biolab, PA) at day-14 of gestation applying 3T9 passaging protocol.

Authentication

NIH3T3 and HEK293T, U2OS, B16F10, A549 and Primary Human Melanocytes cells have the authentication information (STR profiling) from ATCC but they were not independently authenticated by authors. SF9 cells were delivered directly from Gibco but was not independently authenticated by authors. Wistar Institute collection (Philadelphia, PA) delivered melanoma cell lines (1205Lu, WM983B, 451Lu, WM3918, and WM35) authenticated by STR profiling using AmpFlSTR® Identifiler® PCR Amplification Kit (Catalog Number 4322288), no independent authentication was performed by authors. MEFs were identified through genotyping using PCR methods with the primers as follows: Fbxo4, 1loxP forward, 5′-GGCAGAGCTTGAGTTTGCAACATTTCAGGTG-3′, and 3loxP reverse, 5′-TCCTGATCTTT GGAAATTCTTCCTCTGAGT-3′.TE15 cells

	were authenticated by short tandem repeat analysis for highly polymorphic micro-satellites FES/FPS, vWA31, D22S417, D10S526, and D5S592.
Mycoplasma contamination	All cell lines used in the studies were tested negative for mycoplasma contamination.
Commonly misidentified lines (See ICLAC register)	1205LU (Reg. ID: ICLAC-00483)

Animals and other organisms

Policy information about studies involving animals; ARRIVE guidelines recommended for reporting animal research

Laboratory animals	38 (19 male, 19 female) six-week-old C57BL/6J mice used in the study were purchased from The Jackson Laboratory
Wild animals	The study did not involve wild animals.
Field-collected samples	The study did not involve samples collected from the field.
Ethics oversight	The protocol was approved by the Institutional Animal Care and Use Committee (IACUC) at the Case Western Reserve University (CWRU) protocol# 2019-0052

Note that full information on the approval of the study protocol must also be provided in the manuscript.

March 2021
