# Supplementary material for: Tumor suppressor mediated ubiquitylation of hnRNPK is a barrier to oncogenic translation
Source: Nat Commun. 2022 Nov 3;13:6614. doi: 10.1038/s41467-022-34402-6 (PMC9633729; doi:10.1038/s41467-022-34402-6)

Uncropped scans of blots- main figures

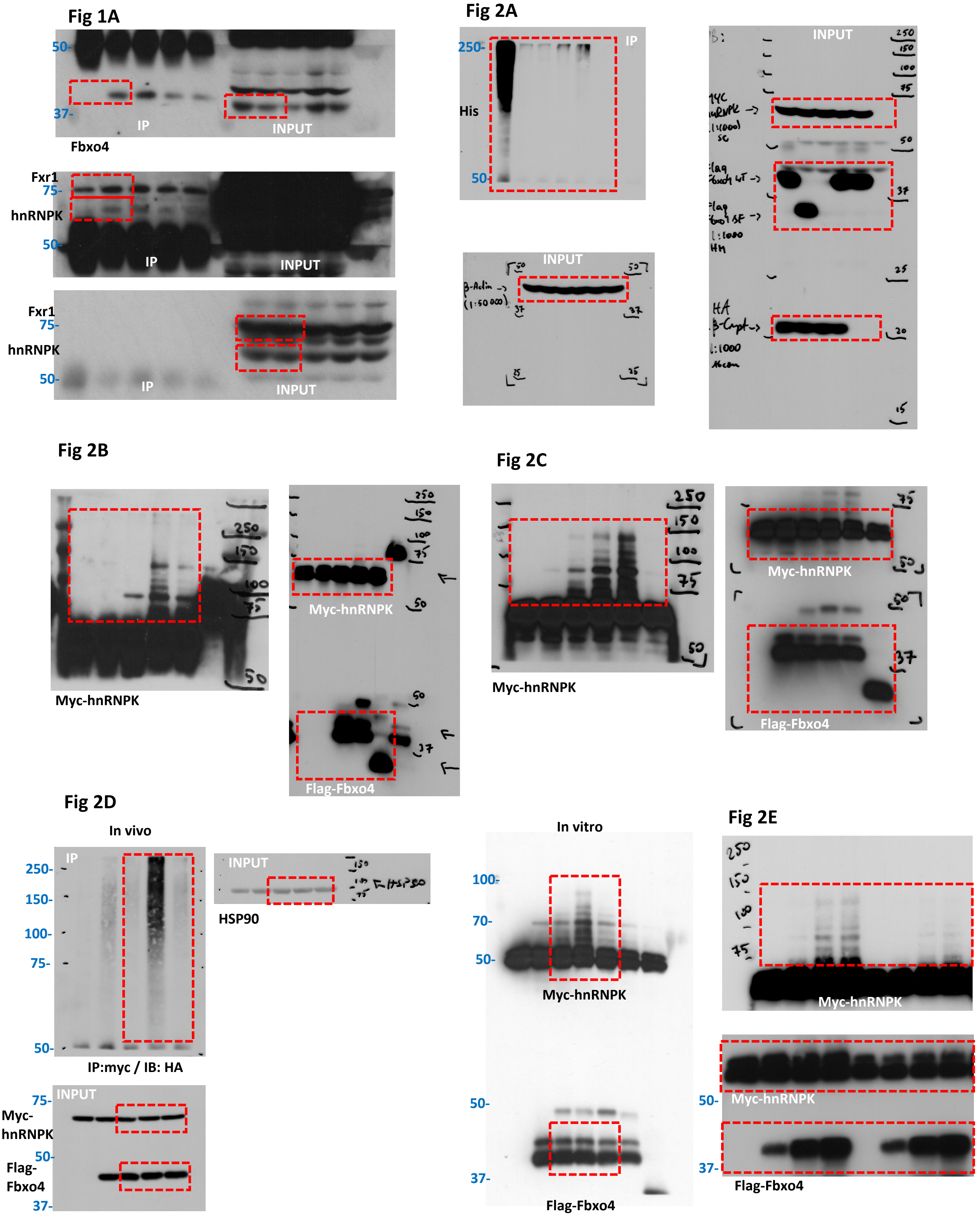

Fig 2F

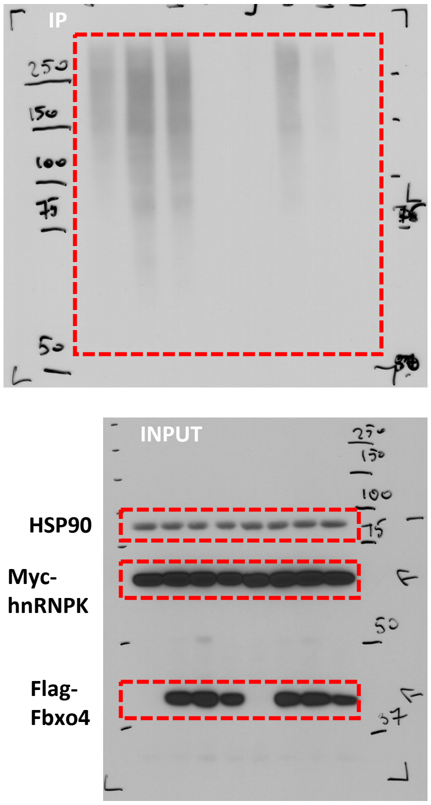

Fig 2H

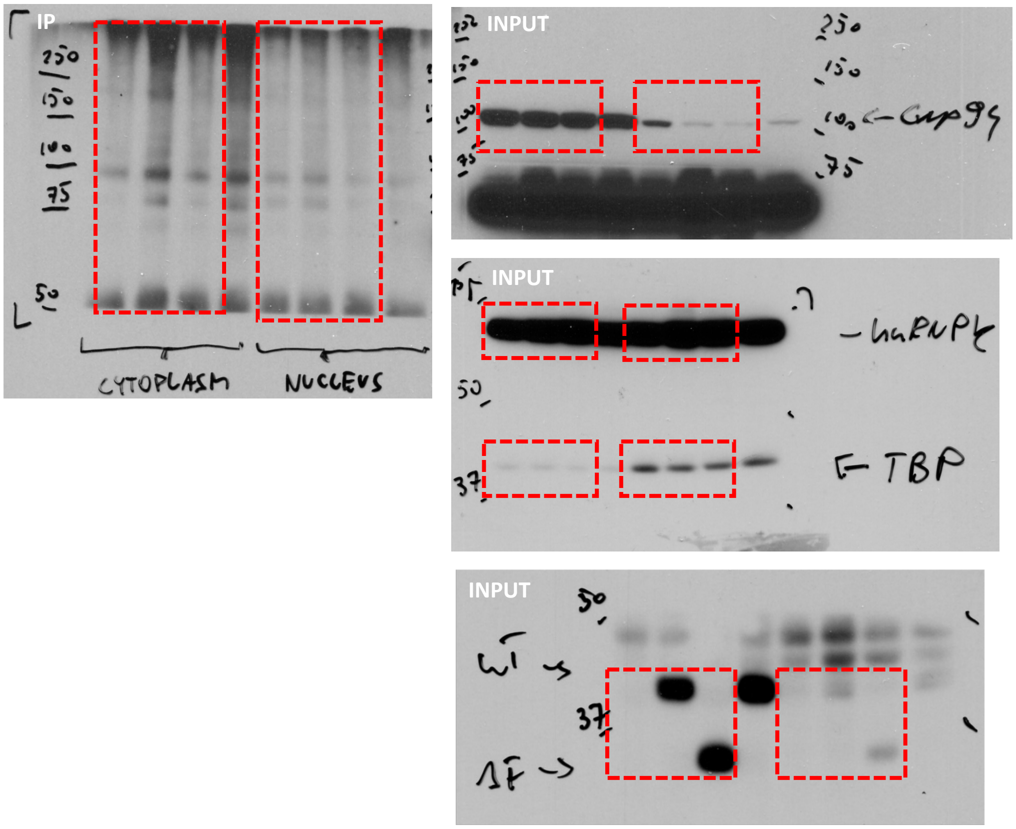

Fig 3B

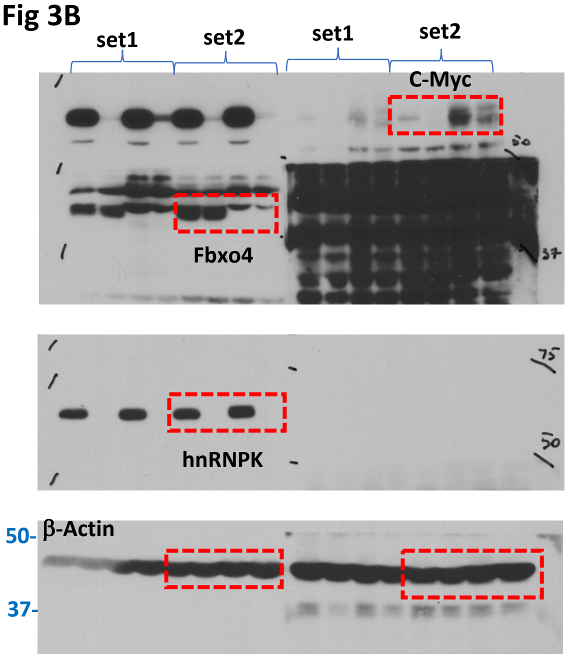

Fig 3C

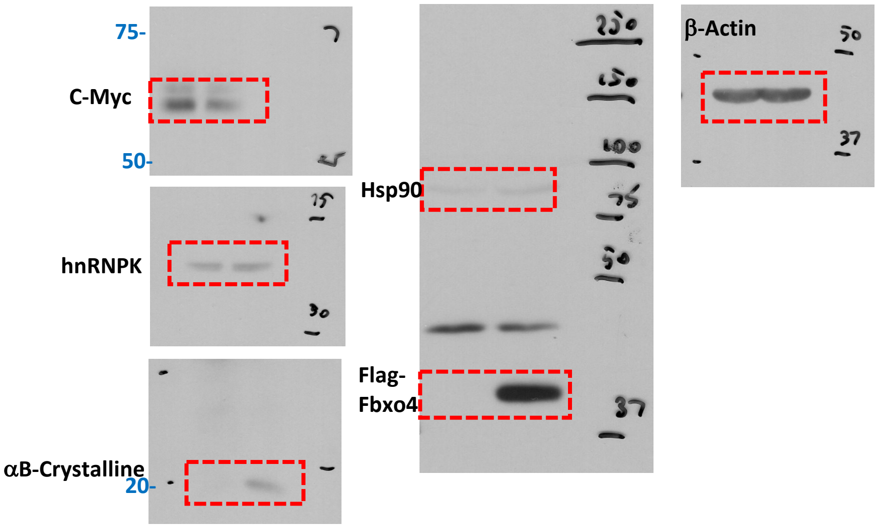

Fig 3K

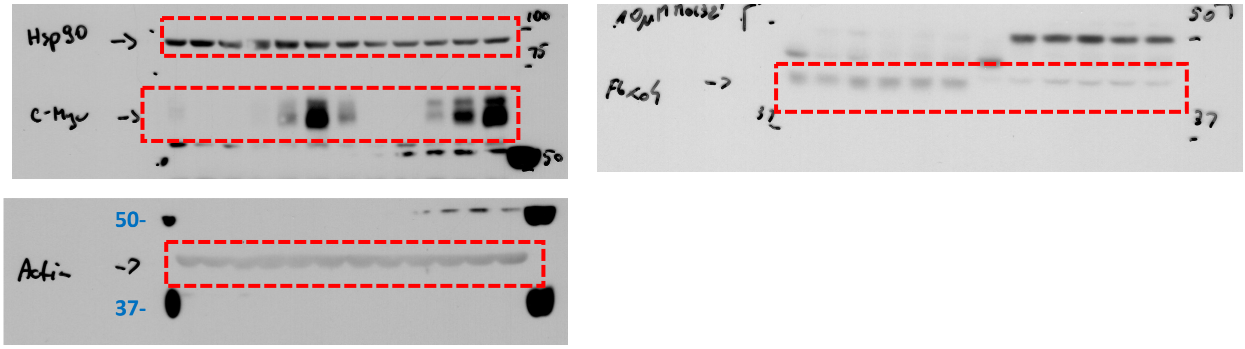

Fig 3L

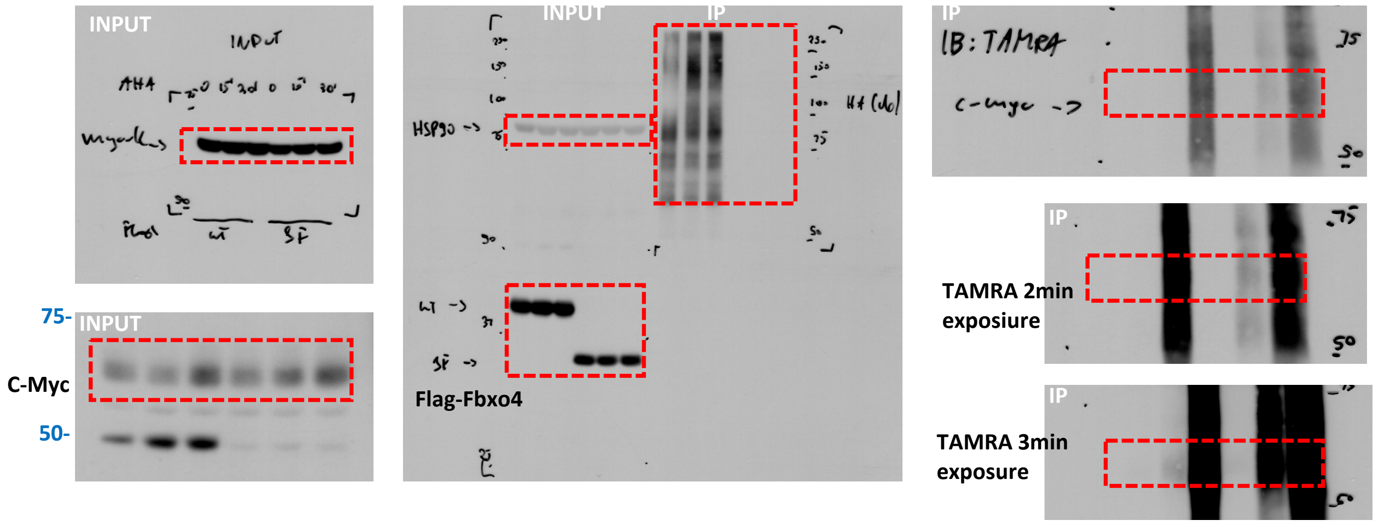

Fig 4E

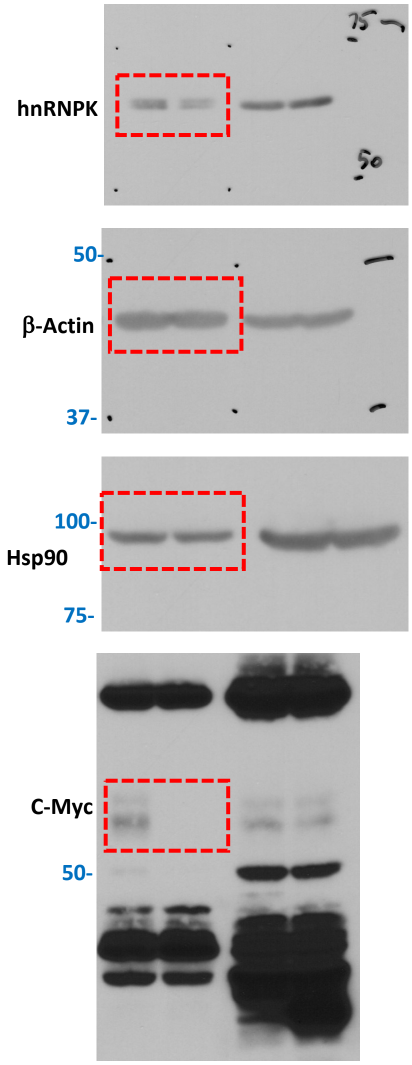

Fig 5A

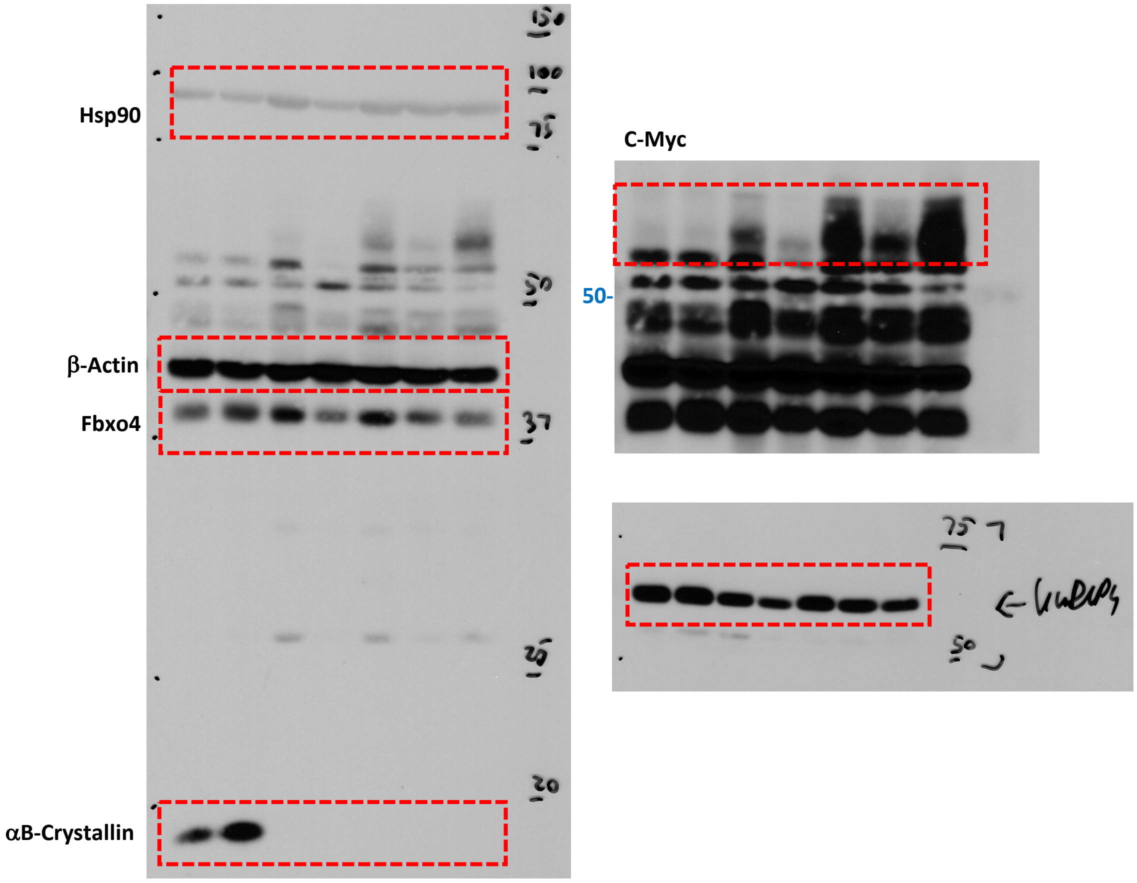

Supplement: Supplementary file 9 — Source Data [file 41467_2022_34402_MOESM9_ESM.zip › Uncropped scans of western blot films main figures.pdf]
