# Supplementary material for: Tumor suppressor mediated ubiquitylation of hnRNPK is a barrier to oncogenic translation
Source: Nat Commun. 2022 Nov 3;13:6614. doi: 10.1038/s41467-022-34402-6 (PMC9633729; doi:10.1038/s41467-022-34402-6)

Uncropped scans of blots - supplementary figures

Fig S1A

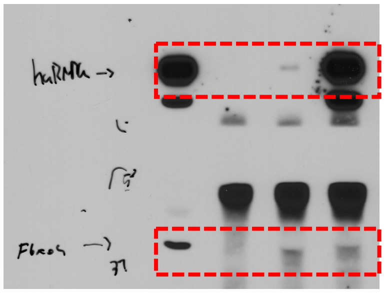

Fig S1B

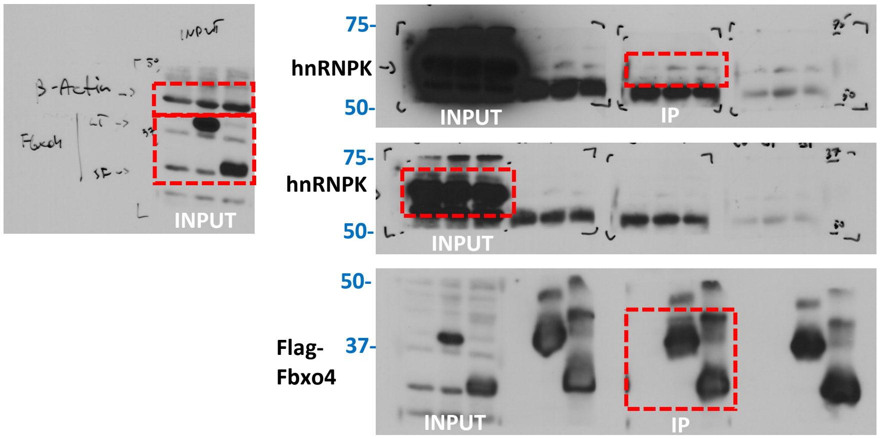

Fig S1D

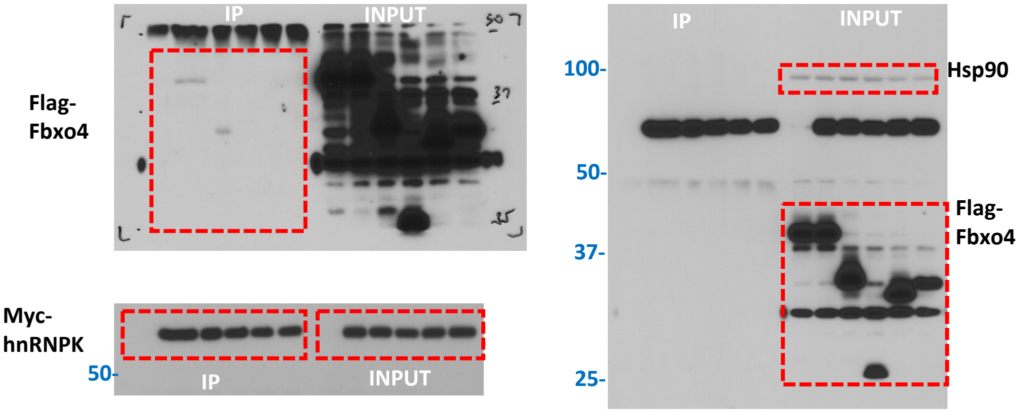

Fig S1E

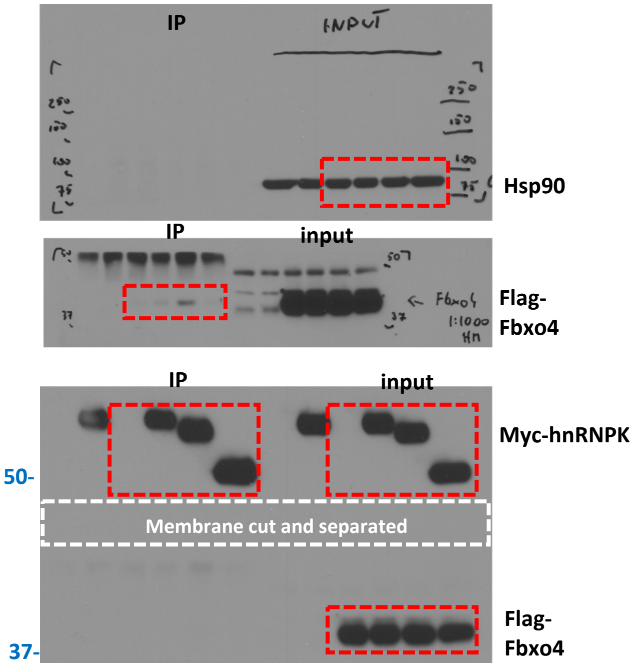

Fig S2A

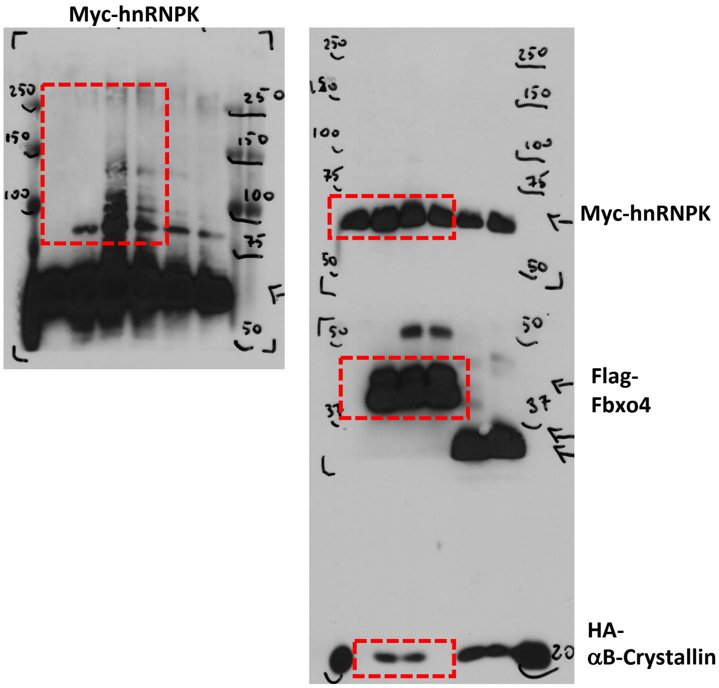

Fig S2C

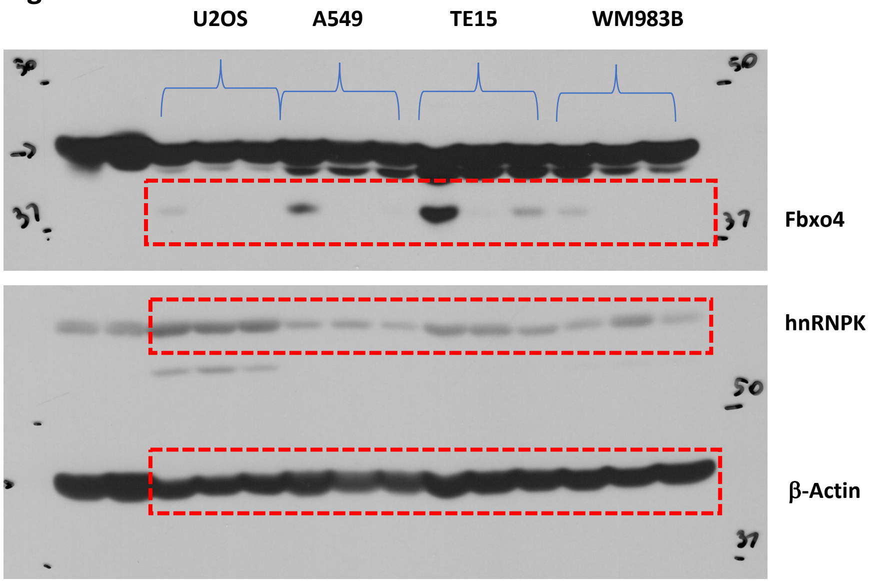

Fig S2D

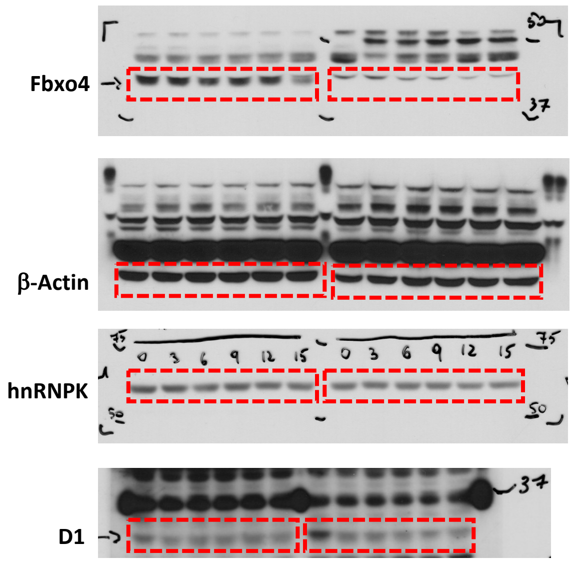

Fig S2E

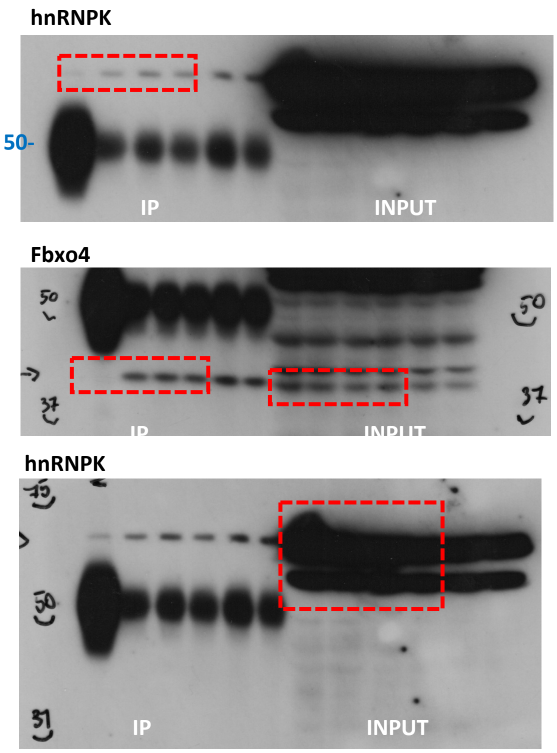

Fig S2F

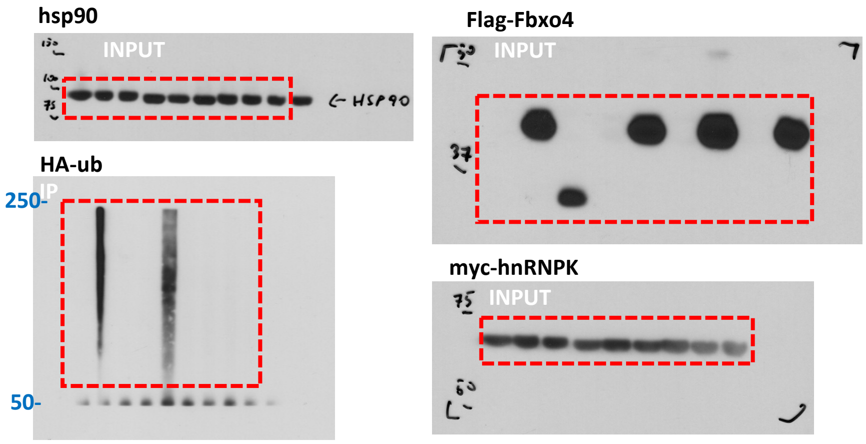

Fig S2H

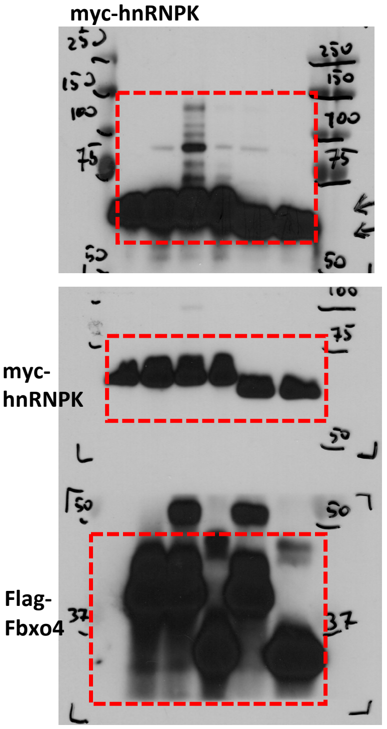

Fig S2J

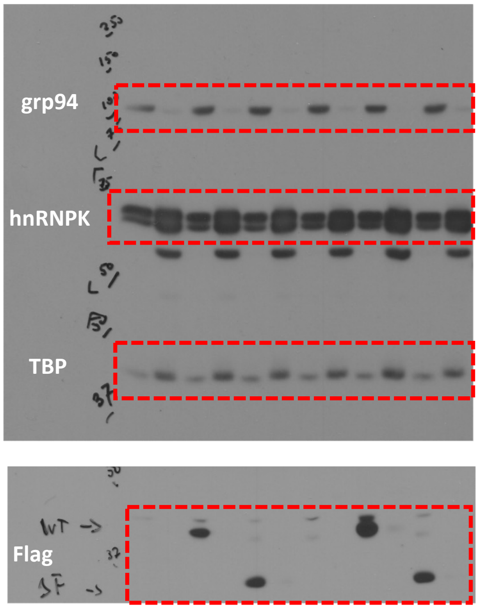

Fig S2I

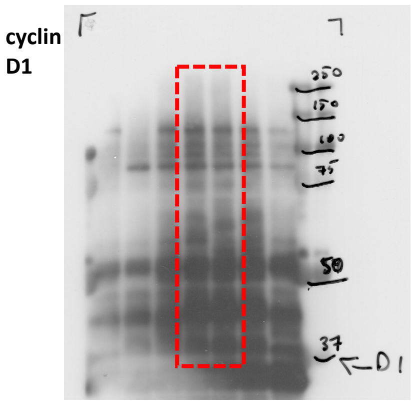

Fig S2K

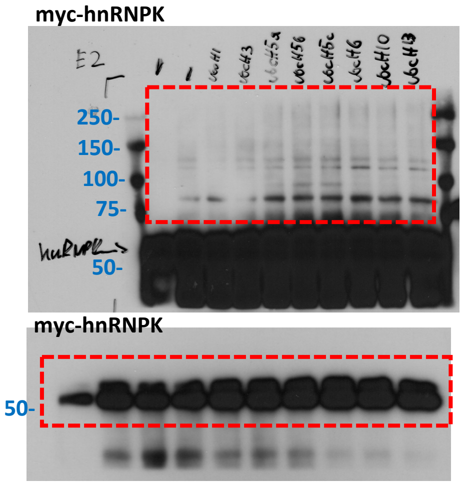

Fig S3B

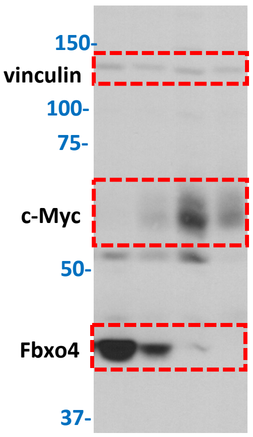

Fig S3D

Fig S3D

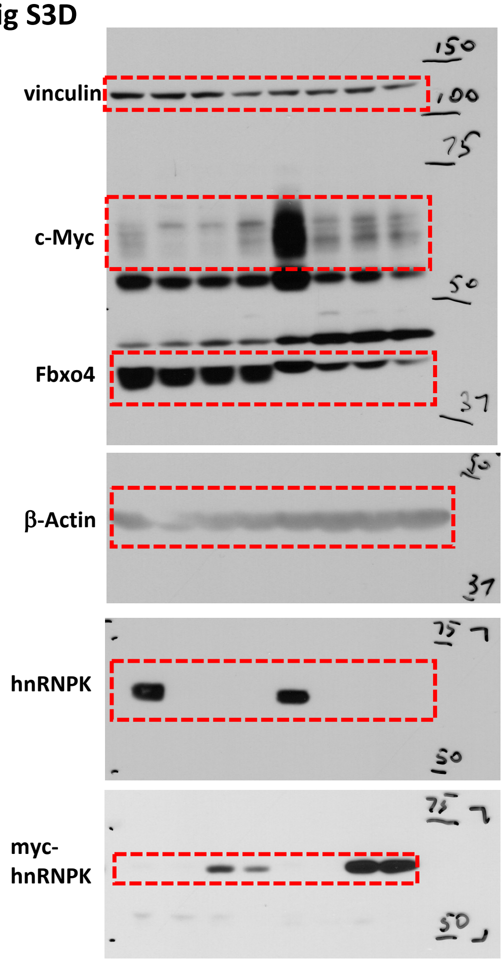

Fig S3G

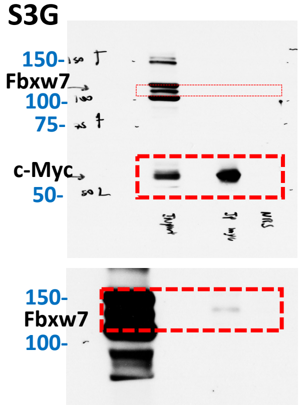

Fig S3E

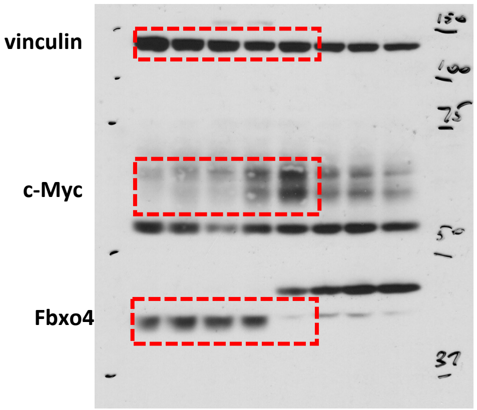

Fig S3L

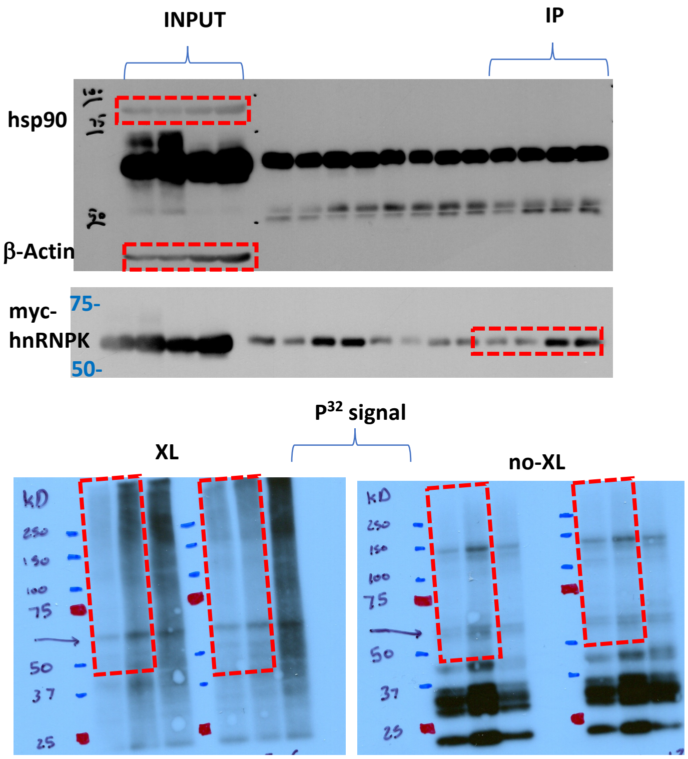

Fig S3H

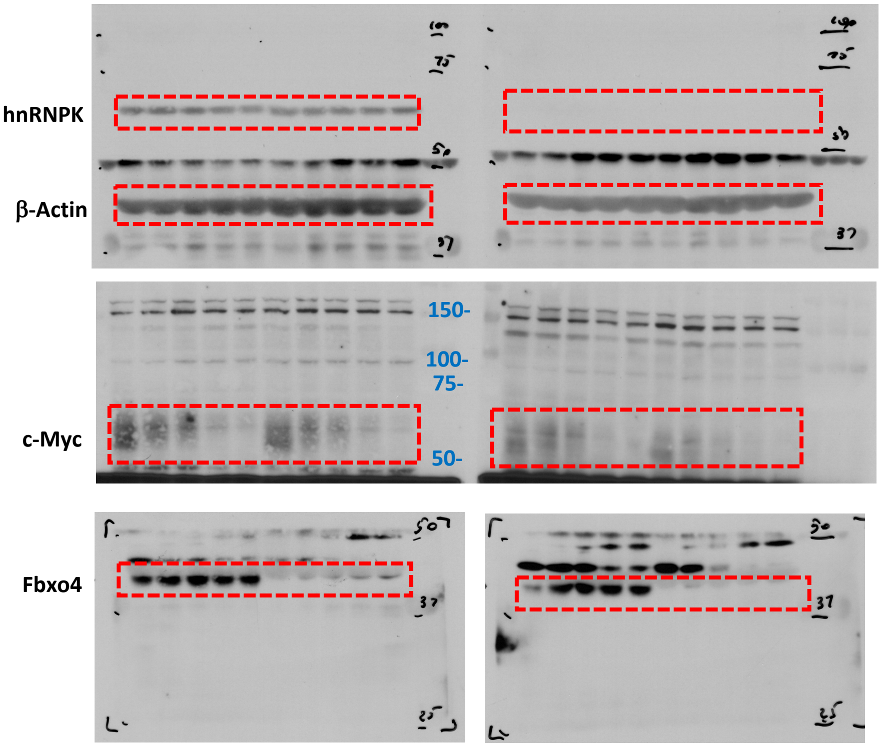

Fig S4E

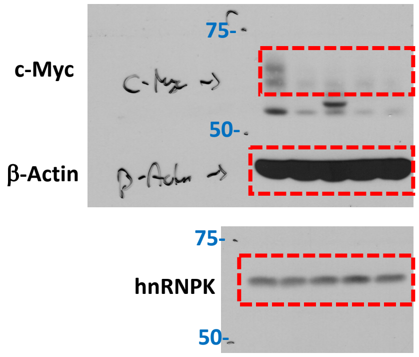

Fig S5A

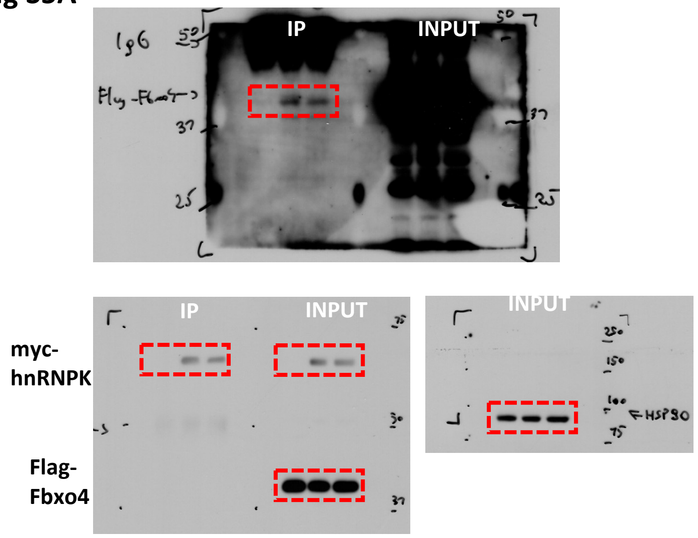

Fig S5B

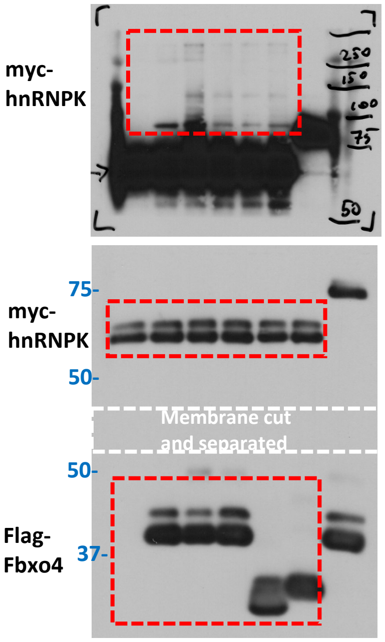

Fig S5C

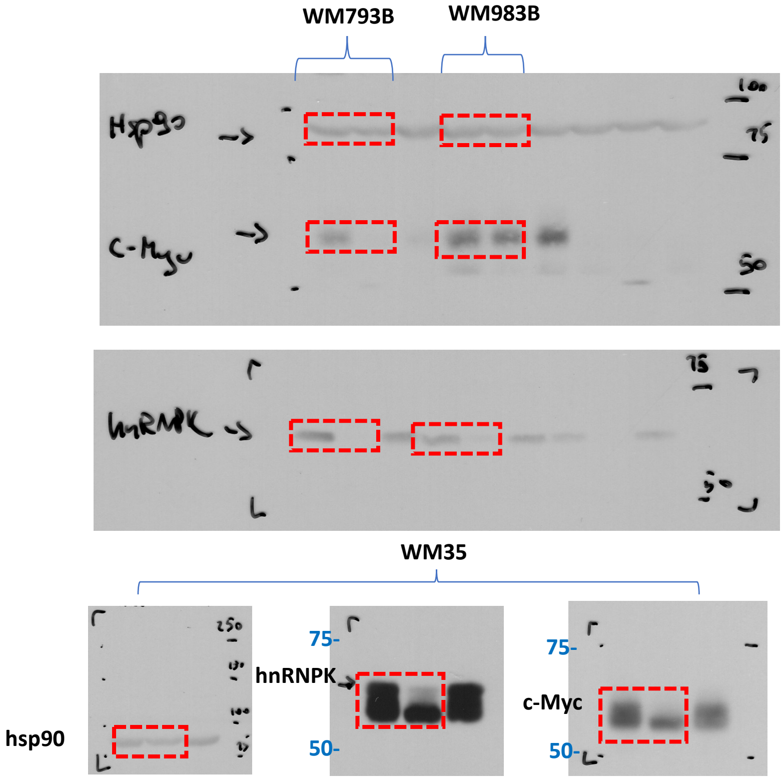

Supplement: Supplementary file 9 — Source Data [file 41467_2022_34402_MOESM9_ESM.zip › Uncropped scans of western blot films supplementary figures.pdf]
